# Solamargine induces apoptosis and ferroptosis through the ROS/p38 MAPK signalling pathway in intrahepatic cholangiocarcinoma

**DOI:** 10.1038/s41598-026-49458-3

**Published:** 2026-04-24

**Authors:** Xiang Wang, Kai Luo, He Bai, Xudong Zhang, Jialin Qu, Xiaoqing Wang, Xu Sun, Yuting Zhao, Bin Wang, Guixin Zhang

**Affiliations:** 1 Department of General Surgery, the Second Hospital of Dalian Medical University, No. 467 Zhongshan Road, Shahekou District, Dalian, 116021 Liaoning China; 2 Clinical Laboratory of Integrative Medicine, First Affiliated Hospital of Dalian Medical University, Liaoning Province, Dalian, 116021 China; 3 Department of Hepatobiliary Surgery, Shandong Provincial Third Hospital, Shandong University, Shandong Province, Jinan, 250031 China; 4 Institute (College) of Integrative Medicine, Dalian Medical University, Liaoning Province, Dalian, 116044 China

**Keywords:** Solamargine, Intrahepatic cholangiocarcinoma, Apoptosis, Ferroptosis, p38 MAPK, Reactive oxygen species, Cancer, Cell biology, Drug discovery, Oncology

## Abstract

Intrahepatic cholangiocarcinoma (ICC) is a highly aggressive liver malignancy with rising global incidence and poor prognosis. Despite recent advances in treatment strategies, effective therapeutic options for ICC remain limited. Solamargine (SM), a natural steroidal alkaloid, has demonstrated anticancer activity, yet its mechanisms in ICC are not fully understood. This study shows that SM inhibits ICC progression by inducing both apoptosis and ferroptosis in vitro and in vivo. SM significantly suppressed proliferation, migration, and invasion of ICC cells (HUCCT-1 and HCCC-9810), with minimal cytotoxicity toward normal biliary epithelial cells (HIBEC). Mechanistically, SM induced mitochondrial dysfunction, ROS accumulation, and lipid peroxidation, leading to apoptosis and ferroptosis. Transcriptome sequencing and gene set enrichment analysis (GSEA) revealed that SM treatment activated apoptosis- and ferroptosis-related pathways, including MAPK signaling. Western blot analysis and pharmacological inhibition assays confirmed that p38 MAPK activation was essential for SM-induced cell death, with ROS acting as an upstream activator. In vivo, SM significantly inhibited tumor growth in both orthotopic and subcutaneous ICC mouse models and showed better tolerability than gemcitabine. Tumor tissue analysis further confirmed increased markers of apoptosis and ferroptosis, along with p38 MAPK activation. These findings suggest that SM exerts dual antitumor effects in ICC, which are associated with the induction of apoptosis and ferroptosis and may involve the ROS/p38 MAPK signaling axis, highlighting its potential as a therapeutic candidate for ICC.

**Supplementary Information:**

The online version contains supplementary material available at 10.1038/s41598-026-49458-3.

## Introduction

Intrahepatic cholangiocarcinoma (ICC) is a primary malignant liver tumor originating from intrahepatic biliary epithelial cells. It accounts for 10%–15% of primary liver cancers and is the second most common primary hepatic malignancy. The global incidence of ICC has been increasing steadily, posing a significant threat to public health^1^. Early-stage cholangiocarcinoma (CCA) patients are typically asymptomatic, and the disease is often diagnosed at an advanced or metastatic stage, exceeding the criteria for surgical resection. As a result, the prognosis remains poor, with a median overall survival (mOS) of only 8 months and a 5-year overall survival (OS) rate of 9%^2^. Over the past decade, the gemcitabine plus cisplatin chemotherapy regimen has been the standard first-line treatment for advanced biliary tract cancer (BTC). Unfortunately, the clinical benefits of these regimens have been limited, with a mOS of approximately one year. For chemotherapy-resistant or relapsed CCA after first-line chemotherapy, treatment options are even more limited.

Natural compounds have gained widespread attention due to their anti-tumor, immune-modulating, multi-target, and low side-effect properties, making them promising candidates for the treatment of various diseases. Solamargine (SM), a natural steroidal alkaloid derived from the traditional Chinese medicine *Solanum nigrum* Linn, has been reported to exhibit multiple biological activities, including anti-cancer effects against cervical cancer, breast cancer, melanoma, liver cancer, and more^3^. It also demonstrates anti-edema, anti-inflammatory, chemosensitizing, and resistance-reversing properties^4^. However, the specific mechanism of SM in anti-ICC remains unclear.

Programmed cell death (PCD) has long been a research hotspot in the field of cancer, as it is a major form of cell death. The most common and extensively studied form of PCD is apoptosis, which plays a critical role in maintaining normal cellular homeostasis^5,6^. It has been established that oxidative stress is a key factor in triggering apoptosis, playing a critical role in tumor initiation, progression, and treatment by promoting the apoptosis of tumor cells. Ferroptosis is a novel form of PCD, characterized by iron overload, ROS accumulation, and lipid peroxidation^7,8^. In recent years, ferroptosis has attracted significant attention in cancer research, demonstrating promising potential in cancer therapy^9^. The progression of ICC is closely related to ferroptosis mechanisms and associated regulatory pathways. ICC cells exhibit a high sensitivity to ferroptosis, where abnormal iron metabolism leads to increased oxidative stress and iron accumulation. Activating ferroptosis to combat cancer represents a safe and selective therapeutic approach. At the same time, there is a correlation between apoptosis and ferroptosis^10^.

The mitogen-activated protein kinases (MAPK) signaling pathway plays a crucial role in oxidative stress and is closely associated with ferroptosis and apoptosis^11^. MAPK is a key regulator of cell proliferation and cancer progression, and studies have shown that the activation of p38 MAPK enhances both apoptosis and ferroptosis^12^. Additionally, the MAPK signaling cascade may be regulated by ROS, which are small, highly reactive molecules that play a central role in regulating physiological processes, maintaining redox balance, and mediating various forms of cell death^13^. Therefore, targeting related signaling pathways, particularly the ROS and p38 MAPK pathways, could represent a promising therapeutic strategy for ICC.

In this study, we demonstrated that SM exhibits anti-tumor potential against ICC. Mechanistic investigations further revealed that SM may induce apoptosis and ferroptosis in ICC via the ROS/p38 MAPK pathway.

## Materials and methods

### Antibodies and reagents

**Fig. 1:**
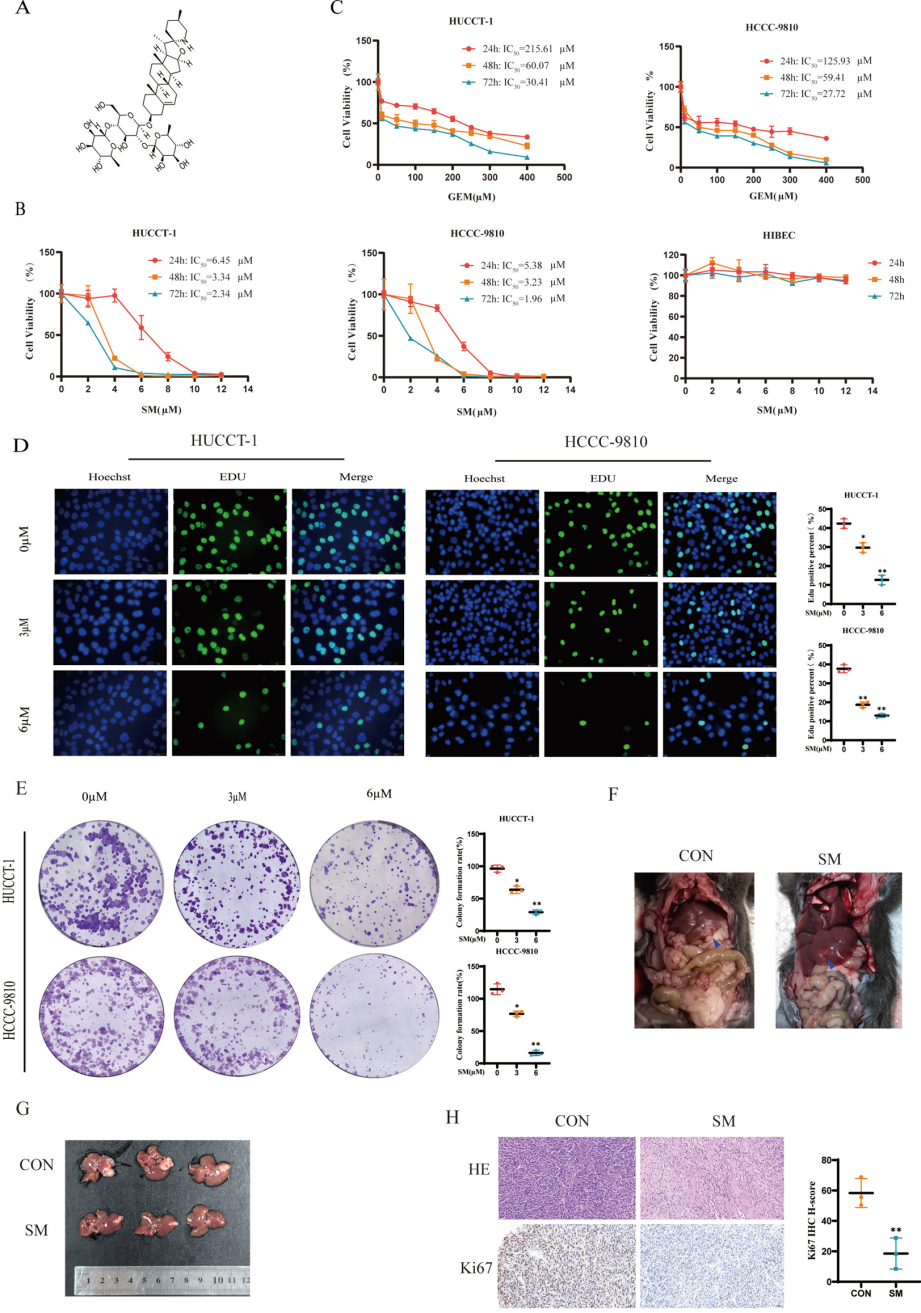
SM can inhibit the proliferation of ICC cells and tumor growth. (**A**) The chemical structure of SM. (**B**) Cell viability of HUCCT-1 and HCCC-9810 (ICC cell lines) and HIBEC (normal biliary epithelial cells) treated with SM (0–12 μM) for 24, 48, and 72 h, as determined by CCK-8 assay. (**C**) The positive control drugs GEM was used to evaluate the inhibitory effect on HUCCT-1 and HCCC-9810 cells. The concentrations of GEM used were 0–400 μM, and the treatment time was 24 h, 48 h and 72 h. (**D**) Presentation of EdU-Positive cell Percentages following 24 h of SM treatment, with the concentrations of SM used were 3 and 6 μM, through the EdU incorporation assay(Scale bars: 50 μm) . (**E**) Colony formation assay was used to detect the effect of SM on the proliferation ability of ICC cells. (**F**) The representative images of livers derived from orthotopic ICC mice after SM treatment (n = 3), blue arrow indicates tumor tissue. (**G**) The tumor tissues at 18 days after the treatment of SM. (**H**) H&E, Ki67 Immunohistochemistry Images in tumor tissues(Scale bars: 50 μm). Data are presented as mean ± standard deviation (SD), with n = 3 for each experimental group. Statistical significance is indicated as *p < 0.05; p < 0.01.

Solamargine was purchased from Aladdin Co., Ltd. (Shanghai, China). The chemical structure is shown in (Fig. 1A). The purity was > 98%. It was dissolved in DMSO to prepare a 20 mM stock solution and diluted with culture medium to final concentrations of 0–12 μM. DMSO was used as the vehicle control, and the final concentration was maintained below 0.1% (v/v) in all experiments. RPMI-1640 medium, 1% penicillin/streptomycin were purchased from Gibco (USA); DMEM medium, fetal bovine serum (FBS) were purchased from Procell (Wuhan, China) ; Gemcitabine (A8437) and Cell counting kit-8 was purchased from APExBio (Houston, United States); Fer-1 (T6500), CQ (T8689), NAC (T0875), Nec-1 (T1847), Z-VAD-FMK (T7020) and SB203580 (T1764) were purchased from Targetmol Chemical Inc. (Shanghai, China); cleaved caspase-3 (#AF7022) were purchased from Affinity Biosciences (OH,USA); Gpx4 (#CY6959) were purchased from Abways (Shanghai,China); Bax (#50,599–2-Ig), Bcl-2 (#12,789–1-AP), Nrf2 (#16,396–1-AP), Slc7a11 (#26,864–1-AP), p38 (#14,064–1-AP), p-p38 (#28,796–1-AP), ERK (#11,257–1-AP), p-ERK (#28,733–1-AP), JNK (#51,151–1-AP), p-JNK (#80,024–1-RR), Beta-actin (#66,009–1-lg), GAPDH (#60,004–1-IG) were purchased from Proteintech (Wuhan, China); EdU-488 Cell Proliferation Kit (SC226) were purchased from Seven/Abcells (Beijing, China); Annexin V-FITC/PI Apoptosis Kit (E-CK-A211) were purchased from Elabscience (Wuhan, China); Mitochondrial membrane potential (MMP) assay kit with JC-1 were purchased from Beyotime Biotechnology (Shanghai, China); MDA Content Assay Kit (BC0025), GSH Content Assay Kit (BC1175), Fe^2+^ Content Assay Kit (BC5415), ROS Assay Kit (CA1410) , Total Protein Extraction Kit (BC3710) were purchased from Solarbio (Beijing, China); Lipid peroxidation assay kits (BODIPY 581/591 C11, S0043), BCA protein assay kits (P0012) were purchased from Beyotime Biotechnology (Shanghai, China).

### Cell culture

The human ICC cells lines, HUCCT-1, HCCC-9810, were purchased from Pricella Biotechnology Co., Ltd.(Wuhan, China); The human intrahepatic biliary epithelial cells line, HIBEC, purchased from bluefbioBiology Technology Development Co., Ltd. (Shanghai, China). The mouse cholangiocarcinoma cells lines, SB-1,were purchased from Yaji Biotechnology Co., Ltd. (Shanghai, China). HUCCT-1, HCCC-9810,were cultured in RPMI-1640 medium; SB-1, HIBEC, were cultured in DMEM medium. All cultures contained 10% fetal bovine serumand 1% penicillin/streptomycin. Cells were maintained at 37℃ with 5% CO_2_.

### Cell counting kit-8 (CCK-8) assay

HUCCT-1, HCCC-9810, and the HIBEC cells (1 × 10^4^cells/well) were inoculated into 96-well plates. Cells were incubated with media containing different concentrations (0, 2, 4, 6, 8, 10,12 μM) of SM for 24, 48, 72 h. The CCK-8 assay was used to evaluate cytotoxicity while 10 μL CCK-8 was added into the medium and incubated for 60 min. The absorbance was measured at 450 nm by the microplate reader (Biotek Cytation™ 3).

### EdU staining assay

The ICC cells (1 × 10^4^cells/well) were inoculated into 96-well plates, and after plating, the cells were treated with SM for 24 h. Cells were transfected with 50 μM EdU working solution (APExBio) for 2 h. Then, the cells were fixed andstained according to the manufacturer’s instructions. The number of EdU-positive cells was determined using a fluorescence microscope (Olympus).

### Colony formation assay

The ICC cells (1 × 10^3^) were grown in 6-well plates and treated with different concentrations of SM (0, 3, 6 μM) for 24 h. The medium containing SM was removed, and the cells were cultured normally for 12–14 days. The colonies were fixed with 4% paraformaldehyde, stained with 0.1% crystal violet solution, washed three times with pure water, air-dried, and photographed.

### Wound healing assay

The ICC cells (4 × 10^5^) were grown in 6-well plates. By the confluence stage, the cells were scratched with a 100 μL pipette tip to make uniform lengths, Subsequently, treated with different concentrations of SM (0, 3, 6 μM). The cells were washed with PBS to remove isolated cell debris, Wound images were taken at 0 and 24 h using microscope (Olympus). Cell migration was quantified using ImageJ.

### Invasion assay

This assays were conducted using transwell inserts with a 6.5-mm, 8.0-µm-pore polyester membrane (Corning). The upper chamber of the transwell apparatus was coated with a diluted Matrigel (1:8). ICC cells (5 × 10^4^) were added to 200uL serum-free RPMI-1640 medium containing different concentration of SM and placed in the upper part of the transwell chamber. The lower chamber was supplemented with 500 uL RPMI-1640 medium containing 20% FBS. Incubated for 48 h in a humidified atmosphere at 37℃ with 5% CO_2_. Non-invading cells on the upper surface of the membrane were gently removed using a cotton swab, while the invading cells in the lower chamber were fixed, stained, and visualized under a microscope (Olympus). The number of invading cells in each group was quantified using ImageJ software.

### Apoptosis analysis

The ICC cells were treated with SM (0, 3, 6 μM) for 24 h. Treated and untreated tumor cells were digested with trypsin, centrifuged, and resuspended in 1 × cold binding buffer. Cell suspensions were supplemented with 5 uL annexin V-FITC and 5 ul propidium iodide (PI) and incubated at room temperature in the dark for a quarter of 20 min. The percentage of apoptotic cells was determined using LSRFortessa™ flow cytometer (BD Biosciences).

### Testing for mitochondrial membrane potential, or MMP

The MMP was evaluated using JC-1 fluorescent probe. The ICC cells were treated with SM (0, 3, 6 μM) for 24 h. Supernatant was collected, and the cells were washed twice with PBS. Subsequently, 1 ml of JC-1 staining working solution was thoroughly mixed with the cells. The cells were then incubated at 37℃ for 20 min in a cell culture incubator. After two washes with JC-1 buffer solution, the cells were supplemented with culture medium and examined and imaged under a fluorescence microscope (Olympus). Fluorescence intensities were measured using ImageJ software.

### RNA extraction and RNA sequencing analysis

HUCCT-1 cells were divided into two groups: control and SM (6 µM) treatment for 24 h. RNA was extracted from HUCCT-1 cells using Trizol method. The RNA samples were sent to LC Bio Technology CO.,Ltd (Hangzhou, China) for further analysis. Differentially expressed genes (DEGs) were identified using DESeq2. Low-expression genes were filtered out, and DEGs were defined as those with an adjusted p-value (FDR) < 0.05 and a fold change (FC) ≥ 1.5 in the SM vs. control comparison. Bioinformatics analyses were conducted using the OmicStudio platform^14^ (https://www.omicstudio.cn/tool).

### Transmission electron microscope (TEM)

HUCCT-1, HCCC-9810 cells were treated with or without SM (6 µM) for 24 h, collected, and fixed with 2.5% glutaraldehyde. TEM imaging was performed by Servicebio (Wuhan, China).

### Reduced glutathione (GSH), malondialdehyde (MDA), ferrous iron (Fe2+) level assay

To determine the intracellular quantities of GSH, a GSH test kit was utilized. The MDA Assay Kit measures the MDA content. Fe^2+^ content was measured using the Cell Ferrous Iron Colorimetric Assay Kit. The detailed measurement was conducted following the manufacturer’s protocol.

### Measurement of the levels of intracellular ROS

In alignment with the manufacturer’s guidelines, cells treated with SM were incubated with a DCFH-DA probe (10 μM) at 37℃ for 20 min. After washing cells with PBS three times, use luorescence microscope (Olympus) to detect the fluorescence intensity of DCF. Fluorescence intensities were measured using ImageJ software .

### Assessment of lipid peroxidation

Lipid peroxidation was detected by BODIPY™ 581/591 C11. In brief, cells, treated with SM for 24 h, BODIPY™ 581/591 C11 (2 µM) was directly incubated in cells that was adherent cultured for 30 min at 37 °C, and then luorescence microscope (Olympus) was used to detect the fluorescence. Fluorescence intensities were measured using ImageJ software.

### Western blotting assessment

Cells with or without treatment were harvested, and the total proteins were extracted in Total Protein Extraction Kit. Measured the protein concentration with the BCA protein assay kit. Proteins were segregated via sodium dodecyl sulfate–polyacrylamide gel electrophoresis (SDS-PAGE) across a 10–12.5% gradient. Subsequent protein transfer was carried out onto polyvinylidene fluoride (PVDF) membranes with a 0.22 μm pore size. After blocking with 5% nonfat milk, membranes were incubated overnight at 4 °C with primary antibodies, followed by secondary antibody incubation. ECL reagents and a Tanon 5200 (Shanghai, China) fully automated chemiluminescence image analysis system were used to visualize the bands. Band intensities were quantified using ImageJ and normalized to the corresponding loading control.

### Animal experiments

Orthotopic tumor model: Six female C57BL/6 mice aged 5-week-old were obtained from the Beijing HFK bioscience Co., Ltd. (Beijing, China). 2 × 10^5^ SB-1 cells were resuspended in 20 ul of PBS mixed with Matrigel (Corning) and injected into the liver. Treatment was initiated 7 days post tumor cell injection. Then, the mice were randomly divided into 2 groups The Control group, the SM group (SM, 4 mg/kg, i.p., once every three days), while the control group was administered with PBS. The mice were euthanized, and tumor samples were collected 18 days after treatment. The excised tumors were carefully dissected and weighed.

Subcutaneous tumor model: Twenty-four female nude mice (BALB/c) aged 5-week-old were purchased from Beijing HFK bioscience Co., Ltd. (Beijing, China). 5 × 10^6^ HUCCT-1 cells were inoculated in the right underarm of the nude mice. After 7 days, when the subcutaneous tumor volume reaches 80–100 mm^3^, the mice are randomly divided into three groups: The Control group, the SM group (SM, 4 mg/kg, i.p., once every three days), and the GEM group ( GEM, 10 mg/kg, i.p., once every three days), while the control group was administered with PBS. Tumor volume was measured every 3 days, and the volume was calculated by the formula: V = l/2 × (length × width^2^). The mice were sacrificed 18 days after intraperitoneal injections of SM and GEM, and the tumors and organs were removed and fixed for staining with H&E and Western blotting.

The animal experiment was approved by the Animal Experiment Ethics Committee of Dalian Medical University, with the registration number AEE24182. All animal procedures were approved by the Institutional Animal Care and Use Committee (IACUC) of Dalian Medical University and conducted in accordance with the ARRIVE guidelines. Mice were anesthetized with isoflurane and euthanized by cervical dislocation at the end of the procedures.

### Hematoxylin and eosin (H&E) staining and immunohistochemistry

Briefly, tumor tissues were fixed with 4% paraformaldehyde, embedded with paraffin, and cut into sections. The thickness is 5 μm. Sections were then performed H&E staining according to standard protocol. Subsequently, the immunohistochemistry analysis was performed using Ki67.

### Terminal deoxynucleotidyl transferase (TdT)-mediated dUTP nick end labeling (TUNEL) assay

The tissue paraffin sections were dewaxed and hydrated, then incubated with Proteinase-K working solution for 20 min. Thereafter, tissues were incubated with a TUNEL reaction mixture and stained with DAPI. TUNEL-positive cells were observed and photographed using a fluorescence microscope.

### Statistical analysis

All experiments were repeated at least three times (n = 3), and the results were expressed as mean values ± standard deviation (S.D.). Then all the data were analyzed by using the One-way analysis of variance (ANOVA) followed by Dunnett’s multiple comparisons tests. *p < 0.05, **p < 0.01, and ***p < 0.001 were considered as significant differences. All statistics were calculated using GraphPad Prism version 8.0.2 software.

## Results

### Effect of SM on ICC cell proliferation and tumor growth

To investigate the effect of SM on the proliferation of ICC cells, we treated ICC cell lines (HUCCT-1 and HCCC-9810) and the HIBEC with SM in a dose- and time-dependent manner. Cell viability was assessed using the CCK-8 assay. The results revealed that SM significantly inhibited the proliferation of ICC cells in a dose- and time-dependent manner, while the survival rate of human normal intrahepatic biliary epithelial cells did not change significantly. Additionally, HCCC-9810 cells were slightly more sensitive to SM than HUCCT-1 cells across all time points (Fig. 1B). We used positive control drugs GEM to treat HUCCT-1 and HCCC-9810 cells, and found that the IC_50_ of positive control drugs was much higher than that of SM (Fig. 1C). An EdU proliferation assay was conducted to investigate SM’s effect on cell proliferation. The results showed that as the concentration of increased, the proliferation of both HUCCT-1 and HCCC-9810 cells significantly inhibited (Fig. 1D). The longterm inhibitory effects of SM on HUCCT-1 and HCCC-9810 cells growth were further evaluated using a colony formation assay. The results demonstrated that as the concentration of increased, the number of colonies formed by HUCCT-1 and HCCC-9810 cells significantly decreased (Fig. 1E). Next, we established an orthotopic mouse model for ICC as a preliminary exploratory validation experiment to initially evaluate the inhibitory effect of SM in vivo. We found that SM significantly inhibited the growth of orthotopic ICC tumor (Fig. 1F-G). H&E and Ki-67 staining demonstrated decreased tumor cell density and proliferation following SM treatment, suggesting an inhibitory effect on tumor growth and increased cell death (Fig. 1H). In conclusion, SM effectively inhibited the proliferation of ICC cells both in vitro and in vivo, while exhibiting minimal toxicity to normal intrahepatic biliary epithelial cells.

### SM inhibits the migration and invasion of ICC cells and induces apoptosis through the mitochondrial pathway

**Fig. 2:**
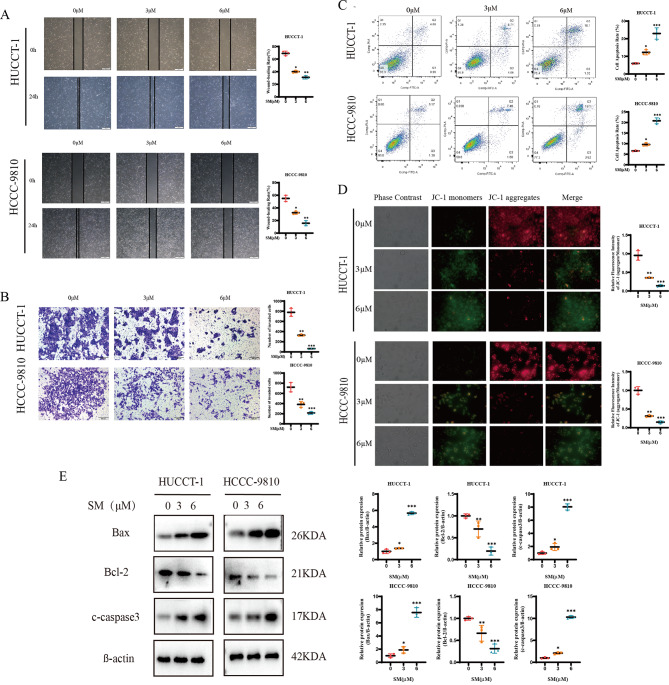
SM inhibits the migration and invasion of ICC cells and induces apoptosis through the mitochondrial pathway. (**A**) Wound healing assays to assess the impact of SM on the migration of HUCCT-1 and HCCC-9810 cells over 24 h ( Scale bars = 500 μm). (**B**) Transwell migration assays (with Matrigel) to evaluate the effect of SM on the invasive capabilities of HUCCT-1 and HCCC-9810 cells, including quantification and representative images (Scale bars = 200 μm). (**C**) Alterations in HUCCT-1 and HCCC-9810 cells post-24-h SM treatment at specified concentrations were monitored by Annexin V-FITC/PI staining, with the percentage of cell death post SM exposure detailed. (**D**) Post-24-h SM treatment in HUCCT-1 and HCCC-9810 cells, shifts in JC-1 staining were observed. (**E**) Protein levels associated with apoptosis in HUCCT-1 and HCCC-9810 cells were evaluated after being subjected to designated SM concentrations 24 h. Data are presented as mean ± standard deviation (SD), with n = 3 for each experimental group. Statistical significance is indicated as *p < 0.05; p < 0.01; *p < 0.001.

Subsequently, we assessed the impact of SM on the migratory and invasive abilities of ICC cells. Accordingly, the wound healing assay was conducted to evaluate cell migration. Our observations demonstrated that different concentrations of SM (0, 3, 6 μM) treatment significantly impeded the migration of HUCCT-1 and HCCC-9810 cells (Fig. 2A). Furthermore, we conducted transwell assays to validate SM’s inhibitory effect on cell invasion. The results demonstrated a significant reduction in the number of invaded HUCCT-1 and HCCC-9810 cells with increased concentration of SM (Fig. 2B). It has been reported that many anticancer drugs exhibit anticancer effects by inducing apoptosis^15^. Apoptosis induction by SM in HUCCT-1 and HCCC-9810 cells was dose-dependent, as demonstrated by flow cytometry (Fig. 2C). Utilizing JC-1 staining, we examined the mechanism behind SM-induced apoptosis in these cells by observing shifts in MMP levels. As the concentration of SM increased, the red fluorescence intensity of mitochondria decreased and the green fluorescence intensity increased significantly in HUCCT-1 and HCCC-9810, suggesting the depolarization of MMP (Fig. 2D), demonstrating that SM triggered mitochondrial dysfunction, leading to apoptosis. The expression of proteins associated with apoptosis was scrutinized through western blot analysis. Increasing SM concentrations corresponded with heightened expressions of Bax, cleaved caspase-3, alongside a reduction in Bcl-2 expression (Fig. 2E). These findings substantiate that SM inhibits the migration and invasion of ICC cells and induces apoptosis through the mitochondrial pathway.

### SM induces changes in the transcription levels of HUCCT-1 cells

**Fig. 3:**
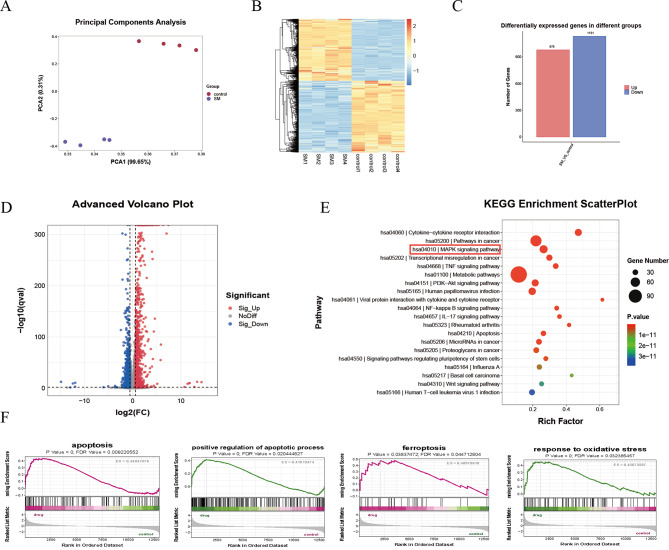
Transcriptome analysis of SM effect in HUCCT-1 cells. (**A**) Principal component analysis between the control and SM (6 μM) groups. (**B**) Heatmap of DEGs between the control and SM groups. Red represents highly-expressed genes, and blue represents low-expressed genes. (**C**) Histogram of the upregulated and downregulated DEGs. (**D**) Volcano plots of DEGs between different groups. (**E**) KEGG enrichment analysis. (**F**) Several representative GSEA results with P-value < 0.05. (n = 4).

For exploring the potential mechanism of the effects of SM on ICC cells, samples were sent for transcriptome sequencing, and RNA-seq was applied to analyze the effect of SM treatment on transcriptional changes in ICC cell lines. HUCCT-1 cells were treated with or without SM(6 μM) for 24 h, and samples were collected for total RNA extraction and sequencing. The principal component analysis and heatmap showed clear distinctions between the control and SM groups (Fig. 3A-B). We detected 2109 DEGs between the control and SM groups. Among the DEGs, 978 genes were upregulated, and 1131 genes were downregulated (Fig. 3C-D). KEGG enrichment analysis based on these obviously altered genes revealed that MAPK pathways was among the most significantly enriched pathways (Fig. 3E). To further explore the potential pathways, we performed GSEA. The representative results were mainly related to pathways such as apoptosis , ferroptosis, Response to oxidative stress (Fig. 3F).

### SM triggers apoptosis and ferroptosis in ICC cells, potentially involving ROS

**Fig. 4:**
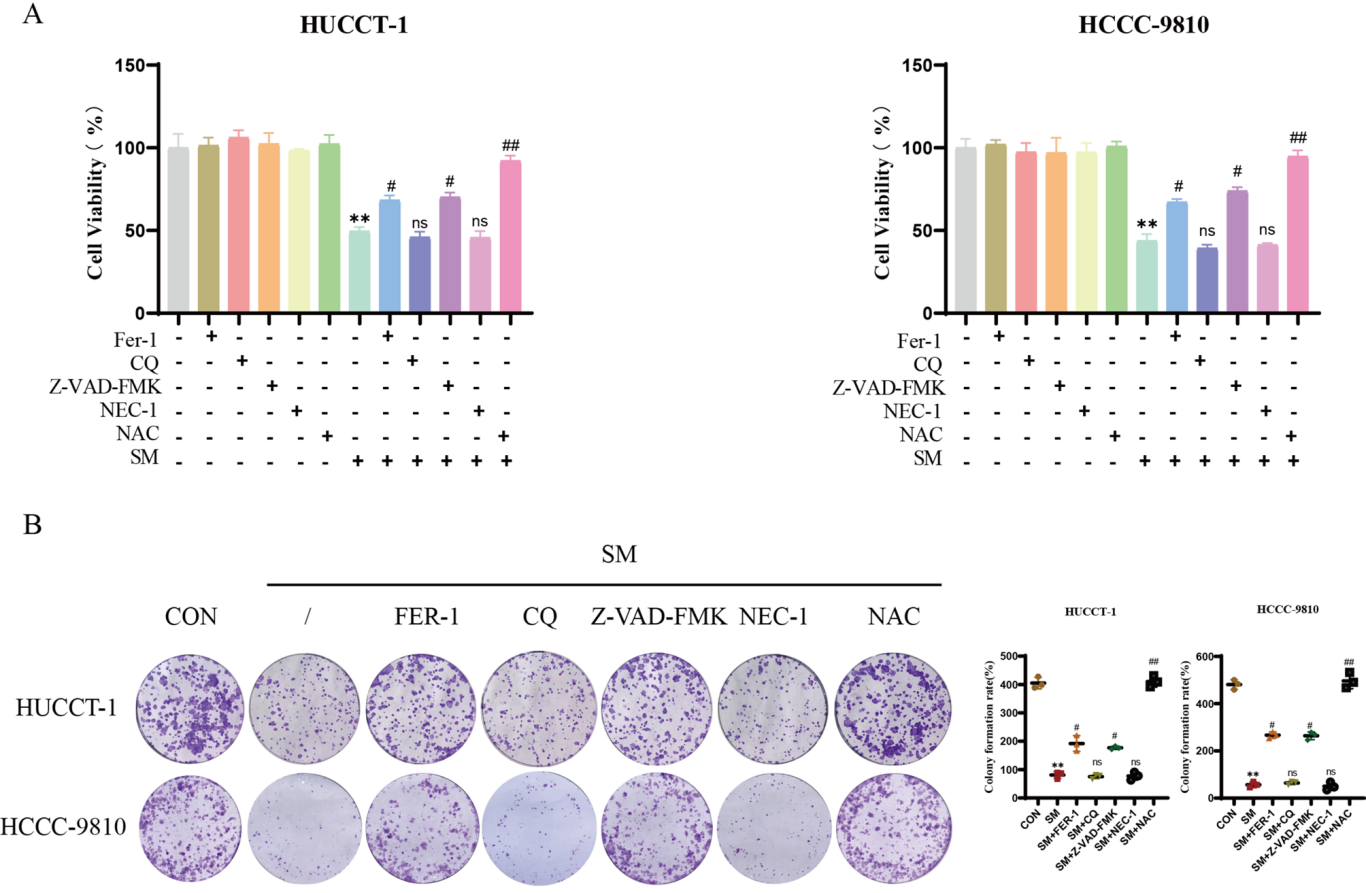
Validation of sequencing results using multiple inhibitors. Cells were precultured with the ferroptosis inhibitor Fer-1(1 μM), autophagy inhibitor CQ(10 μM) , apoptosis inhibitor Z-VAD-FMK(10 μM), necroptosis inhibitor Nec-1(10 μM ), ROS inhibitor NAC (5 mM) for 1 h and then exposed to SM (6 μM) for 24 h. (**A**) Cell viability was used to assessed using the CCK-8 assay. (**B**) Colony formation experiment was used to evaluate the proliferative capacity. Data are presented as mean ± standard deviation (SD), with n = 3 for each experimental group. Statistical significance is indicated as P < 0.01 in comparison to the control cohort. #P < 0.05, ##P < 0.01 in comparison to the SM cohort.

To investigate the forms of cell death caused by SM on ICC cells, a range of inhibitors was employed. we pretreated ICC cells with Fer-1 (ferroptosis inhibitor, 1 μM ), CQ (autophagy inhibitor, 10 μM) , Z-VAD-FMK (apoptosis inhibitor, 10 μM), Nec-1 (necroptosis inhibitor, 10 μM ), NAC (scavenger of ROS, 5 mM) for 1 h, and then incubated the cells with SM (6 µM) for 24 h. The CCK-8 assay findings indicated that the diminished viability of ICC cells treated with SM was restored by inhibitors of apoptosis and ferroptosis, while inhibitors of necroptosis and autophagy did not have any impact. Moreover, the enhancement of viability within ICC cells exposed to SM was more noticeable after NAC treatment, as depicted in (Fig. 4A). The colony formation assay also confirmed this (Fig. 4B). In summary, these results indicated that SM could induce cell apoptosis and ferroptosis of ICC cells and has a strong association with the accumulation of ROS.

### SM induces ferroptosis in ICC cells

**Fig. 5:**
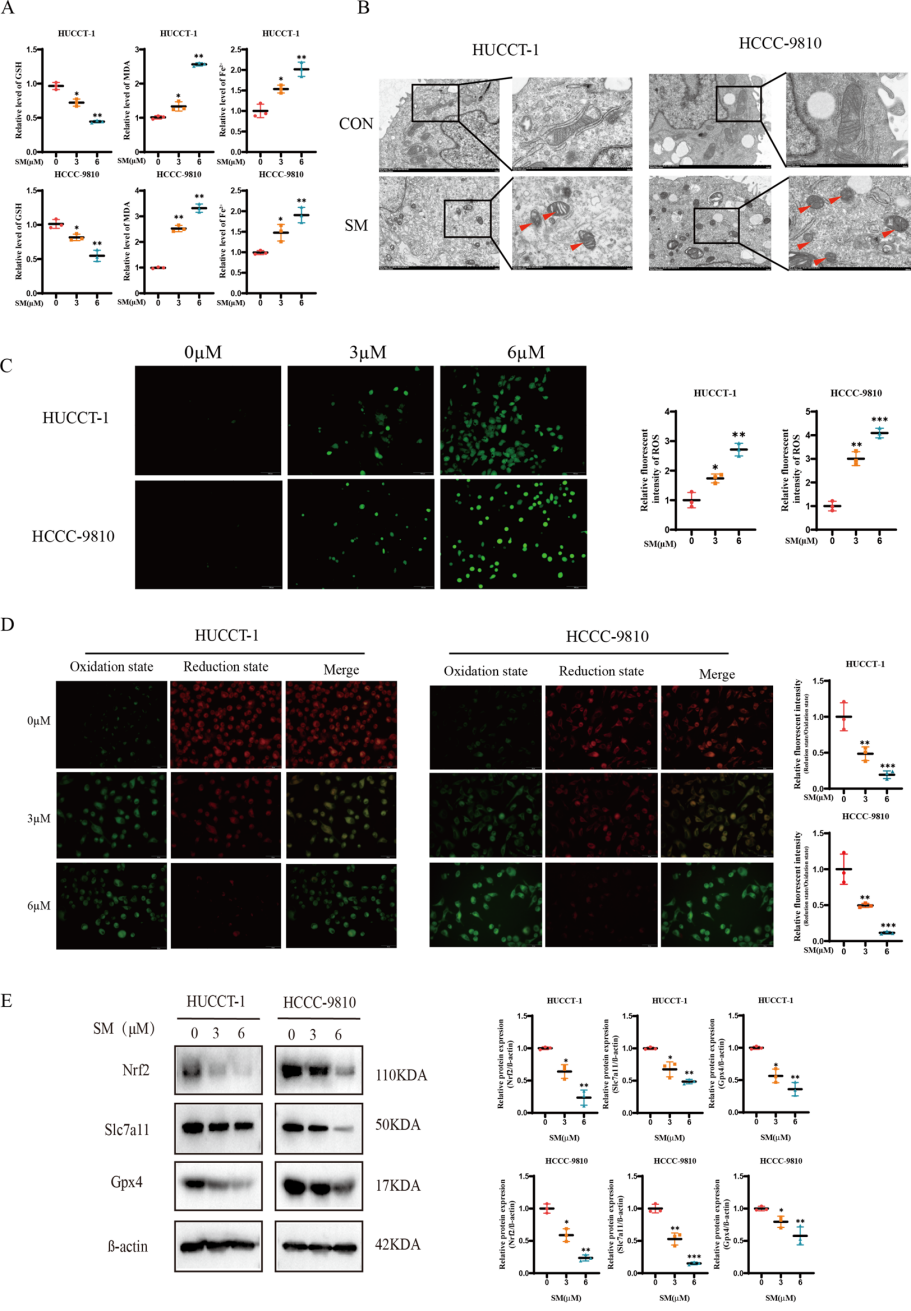
SM Induced ferroptosis in ICC cells. (**A**) GSH, MDA, and ferrous iron assays were performed on HUCCT-1 and HCCC-9810 cells following a 24 h treatment with SM. (**B**) Morphological changes in HUCCT-1 and HCCC-9810 cells exposed to SM(6 µM) for 24 h were observed by transmission electron microscopy (Scale bar, 2 μm and 500 nm). The red arrowheads indicate damaged mitochondria. (**C**) HUCCT-1 and HCCC-9810 cells were exposed to SM for 24 h and then stained with DCFH-DA for 20 min. ROS generation was observed by fluorescence microscopy and representative images are presented (Scale bar, 100 μm). (**D**) Lipid peroxidation levels in SM-treated HUCCT-1 and HCCC-9810 cells were assessed using BODIPY™ 581/591 C11 staining and visualized by fluorescence microscopy (Scale bar, 50 μm). (**E**) Western blotting was used to detect the expression of Gpx4, Slc7a11 and Nrf2 in HUCCT-1 and HCCC-9810 cells upon exposure to SM. Data are presented as mean ± standard deviation (SD), with n = 3 for each experimental group. Statistical significance is indicated as *p < 0.05; p < 0.01; *p < 0.001.

The GSEA enrichment analysis indicated that SM affects HUCCT-1 cells not only through apoptosis but also through ferroptosis. Additionally, the viability of HUCCT-1 and HCCC-9810 cells significantly decreased following treatment with SM. This effect was notably reduced by Fer-1. To clarify whether SM induces ICC cells to undergo ferroptosis, we tested changes in GSH, lipid peroxidation and ferrous iron. In HUCCT-1 and HCCC-9810 cells, SM treatment for 24 h significantly decreased intracellular GSH levels while increasing lipid peroxidation (MDA) and intracellular ferrous iron (Fig. 5A). Furthermore, transmission electron microscopy (TEM) analysis revealed significant mitochondrial morphological changes following SM (6 µM) treatment for 24 h. In the control group, mitochondria exhibited well-defined fusiform structures with intact cristae and bilayer membranes. In contrast, mitochondrial damage was severe in the SM-treated group, characterized by mitochondrial shrinkage, blurred mitochondrial membranes, increased matrix density, and cristae expansion, fragmentation and reduction. These changes are consistent with the mitochondrial features of ferroptosis (Fig. 5B). Ferroptosis is a form of ROS-dependent cell death driven by lipid peroxidation and closely associated with intracellular ROS levels. SM-treated ICC cells were stained with DCFH-DA to assess ROS generation. The results showed that, SM induced ROS generation in a dose-dependent manner (Fig. 5C). Staining images obtained using the C11-BODIPY 581/591 fluorescence probe showed increased green fluorescence in ICC cells after being treated with SM (Fig. 5D), indicating greater lipid ROS production in these cells. In the end, western blotting was used to conduct protein expression assays for molecules related to ferroptosis. The levels of Nrf2, Gpx4, Slc7a11 decreased as the SM concentration increased (Fig. 5E).

### SM inhibits ICC cells through the p38 MAPK pathway

**Fig. 6:**
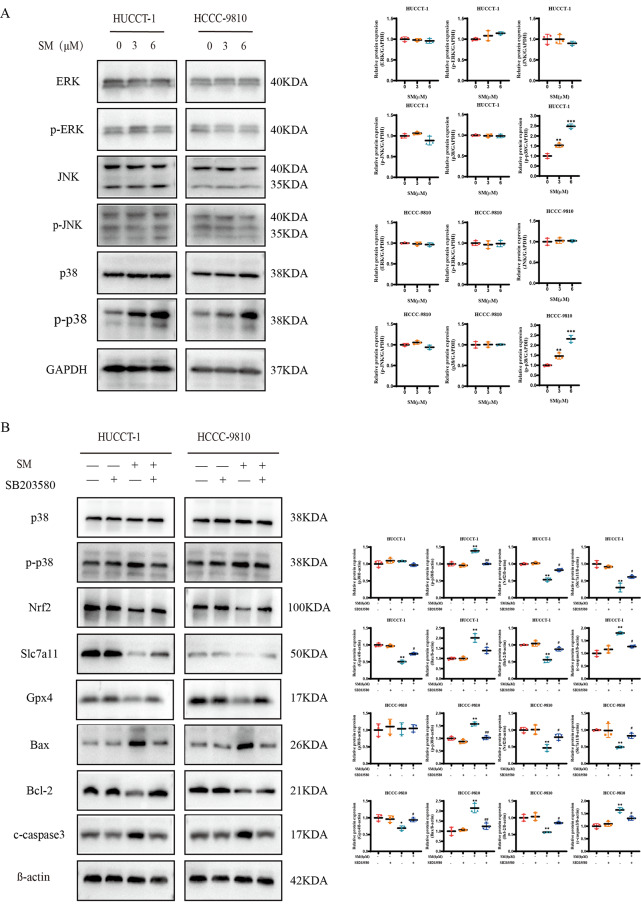
SM inhibits ICC cells through the p38 MAPK pathway. (**A**) The expression levels for MAPK-related proteins in ICC cells were assessed after treatment with different concentrations of SM with a duration of 24 h. (**B**) ICC cells were precultured with p38 MAPK inhibitor SB203580 (10 μM) for 1 h and then exposed to SM (6 μM) for 24 h. The expression levels of Bax, Bcl-2, cleaved caspase-3, Gpx4, Nrf2, Slc7a11 and p-p38 were analysed by western blotting. Data are presented as mean ± standard deviation (SD), with n = 3 for each experimental group. Statistical significance is indicated as *P < 0.05, P < 0.01, *p < 0.001 in comparison to the control cohort. #P < 0.05, ##P < 0.01 in comparison to the SM cohort.

KEGG enrichment analysis of our transcriptome data revealed significant enrichment in the regulation of MAPK cascades. The activation of the MAPK signaling pathway has been implicated in cancer cell death^16^. Based on this, we investigated MAPK activation in HUCCT-1 and HCCC-9810 cells following SM treatment. Western blot analysis was performed to assess the total and phosphorylated levels of p38, JNK, ERK. SM treatment upregulated the phosphorylation of p38 in a dose-dependent manner (Fig. 6A), while its effects on JNK and ERK phosphorylation were minimal. Moreover, precultured with the p38 MAPK inhibitor SB203580 suppressed SM-induced activation of apoptosis- and ferroptosis-related proteins (Fig. 6B). These findings suggest that the p38 MAPK pathway plays a crucial role in SM-induced apoptosis and ferroptosis.

### SM-induced ROS initiates apoptosis and ferroptosis in ICC cells through the ROS/p38 MAPK pathway

**Fig. 7:**
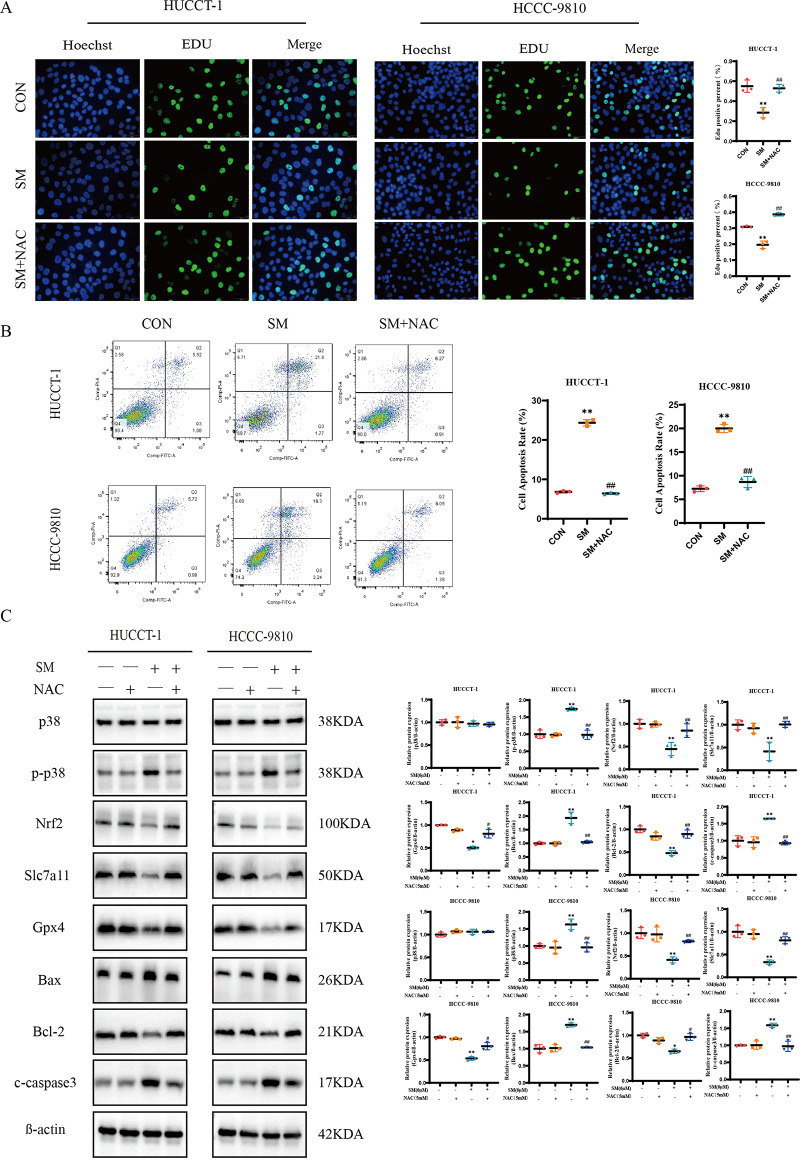
The generation of ROS by SM suppressed cell proliferation and induced cell death. ICC cells were precultured with the ROS inhibitor NAC (5 mM) for 1 h and then exposed to SM (6 μM) for 24 h. (**A**) Cell proliferation was assessed using the EdU incorporation assay(Scale bars: 50 μm) . (**B**) The apoptotic proportion was measured by flow cytometry. (**C**) The expression levels of Bax, Bcl-2, cleaved caspase-3, Gpx4, Nrf2, Slc7a11 and p-p38 were analysed by western blotting. Data are presented as mean ± standard deviation (SD), with n = 3 for each experimental group. Statistical significance is indicated as *P < 0.05, P < 0.01 in comparison to the control cohort. #P < 0.05, ##P < 0.01 in comparison to the SM cohort.

The decreased viability for the HUCCT-1 and HCCC-9810 cells caused by SM was partially restored by Fer-1 and Z-VAD-FMK,whereas it was almost entirely prevented by NAC. Cells were pretreated with NAC (5 mM) for 1 h, followed by EdU incorporation assay and flow cytometry to assess cell proliferation and apoptosis, respectively. The EdU assay revealed that NAC significantly reversed the SM-induced reduction in EdU-positive cells (Fig. 7A). Similarly, flow cytometry analysis demonstrated that NAC markedly attenuated the increase in apoptosis caused by SM treatment (Fig. 7B). Moreover, western blot analysis showed that NAC effectively suppressed SM-induced activation of apoptosis- and ferroptosis-related proteins while strongly inhibiting p38 phosphorylation (Fig. 7C). These results revealed that ROS may be the important factor upstream of p38 MAPK that initiates SM-induced apoptosis and ferroptosis.

### SM inhibits ICC in vivo

**Fig. 8:**
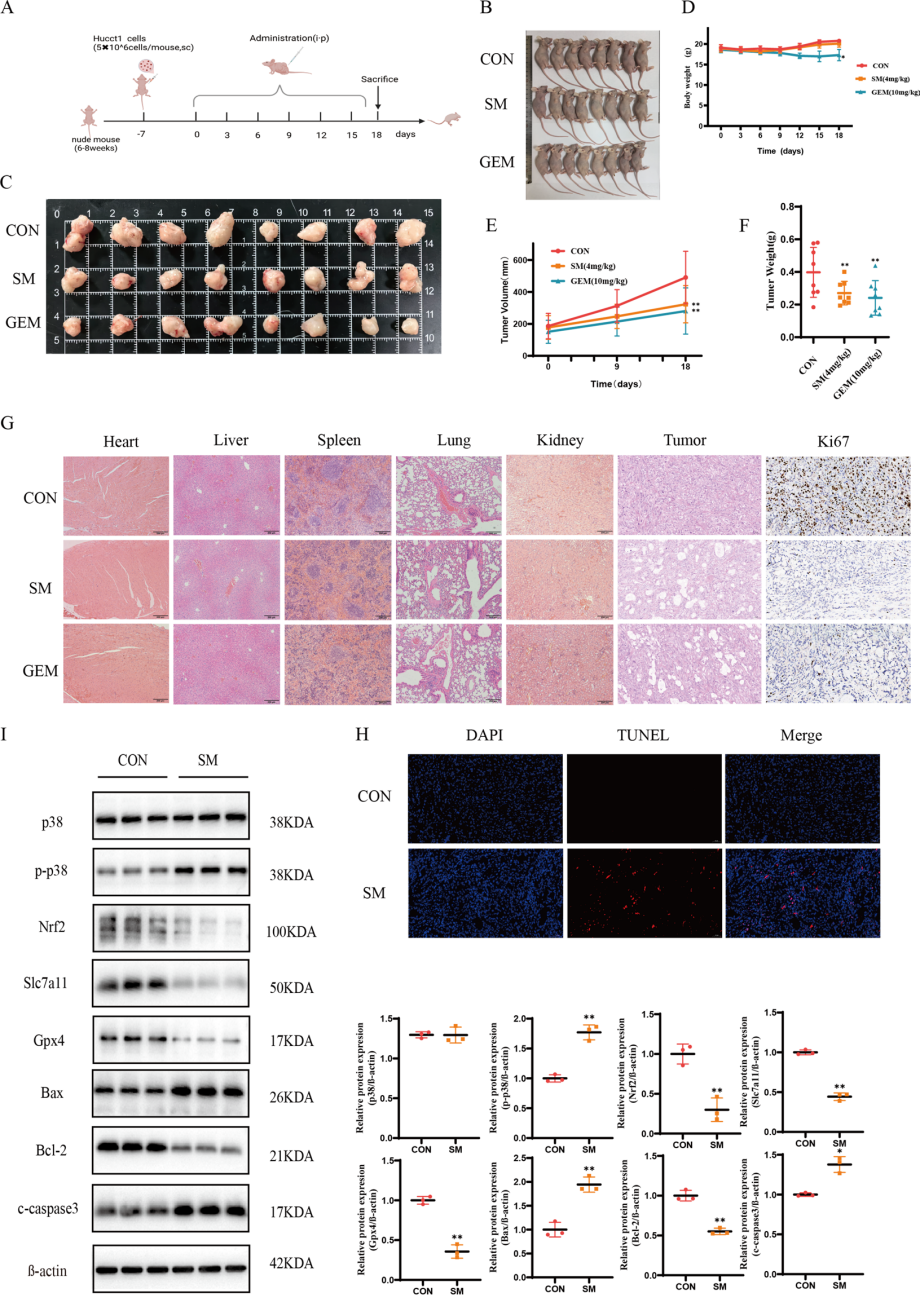
SM inhibits the growth of human ICC xenografts in vivo. (**A**) Experimental set-up: BALB/c mice were subcutaneously injected with HUCCT-1 cells and treated with SM (4 mg/kg, every 3 days, i.p.) and GEM (10 mg/kg, every 3 days, i.p.). (**B,C**) Images of mice and resected tumors at the end of the experiment. (**D**) The growth curve of body weight during treatment with SM and GEM. (**E)** The weight of tumor tissues at 18 days after treatment with SM and GEM. (**F**) The growth curve of tumor volume during treatment with SM and GEM. (**G**) H&E staining was used to observe the morphology of the vital organs(Scale bars: 200 μm) and tumor(Scale bars: 50 μm) ; Immunohistochemical was used to observe the expression of Ki67 proteins(Scale bars: 50 μm) . (**H**) The apoptotic status of tumour tissues was assessed by TUNEL assays(Scale bars: 50 μm). (**I**) The levels of Bax, Bcl-2, cleaved caspase-3, Gpx4, Nrf2, Slc7a11, and p-p38 were analysed by western blotting. Data are presented as mean ± standard deviation (SD), with n = 3 for each experimental group. Statistical significance is indicated as *p < 0.05; p < 0.01.

To better observe the above anti-cancer effect of SM, a subcutaneous HUCCT-1 xenograft tumor assay was further carried out (Fig. 8A-C). Compared to the control group, SM treatment did not result in significant changes in body weight. In contrast, the body weight of mice in the GEM-treated group was lower than that in both the SM-treated group and the control group, suggesting that SM may have a milder or less toxic effect (Fig. 8D). Moreover, SM could significantly inhibit HUCCT-1 xenograft tumor growth, evidenced by dampened increases in tumor weight and volume (Fig. 8E-F). H&E staining, Ki-67 immunohistochemistry, and TUNEL assay revealed that SM treatment reduced tumor cell proliferation and increased tumor cell death, with no apparent organ toxicity in nude mice (Fig. 8G-H). Finally, to further confirm the regulatory effects of SM on key signaling pathways, Western blot analysis was performed on mouse tumor tissues. Similar to in vitro study, the Western blot results showed that, following SM treatment, there was an increase in p38 phosphorylation, along with the activation of apoptosis-related proteins (upregulation of cleaved caspase-3 and Bax, downregulation of Bcl-2) and ferroptosis-related proteins (downregulation of Nrf2, Gpx4, and Slc7a11) (Fig. 8I). In summary, SM induces apoptosis and ferroptosis by activating the p38 signaling pathway, thereby inhibiting the progression of ICC, with a lower incidence of toxicity.

## Discussion

Intrahepatic cholangiocarcinoma (ICC) is a distinct and aggressive malignancy that differs from perihilar and distal cholangiocarcinoma, as well as gallbladder cancer. Due to its insidious onset and lack of early symptoms, ICC is often diagnosed at an advanced stage, at which point surgical resection is no longer a viable option. Although significant progress has been made in molecular targeted therapy and immunotherapy, their clinical efficacy remains limited, and several therapeutic strategies are still under investigation^17^. Therefore, there is an urgent need to identify and develop novel therapeutic agents for ICC.

Solamargine (SM), a bioactive compound derived from the traditional Chinese herb *Solanum nigrum*, has demonstrated potent anticancer properties and is considered a promising antitumor agent^18^. However, its therapeutic potential in ICC remains unclear. In this study, we investigated the inhibitory effects of SM on HUCCT-1 and HCCC-9810 cholangiocarcinoma cells. We assessed cytotoxicity, colony formation ability, and migratory potential using cytotoxicity assays, plate colony formation assays, and wound-healing assays, respectively. Additionally, our findings demonstrated that SM exhibited no significant toxicity toward normal intrahepatic bile duct cells within the tested concentration range.

To investigate the potential mechanism of SM on HUCCT-1 cells, RNA-Seq were used to analyze gene changes at the transcriptional level. we discovered that the differentially expressed genes were significantly enriched in MAPK signal. Further GSEA enrichment analysis of the differentially expressed genes revealed pathways related to apoptosis, response to apoptosis, ferroptosis, and oxidative stress.

Apoptosis is a fundamental mechanism of drug-induced cell death and is often referred to as programmed cell death. Extensive research has demonstrated the pro-apoptotic effects of SM in various cancer models^19,20^. In this study, Annexin V-FITC/PI staining and the application of the pan-caspase inhibitor Z-VAD-FMK confirmed that SM-induced apoptosis in ICC cells is caspase-dependent, underscoring the pivotal role of mitochondria in this process. Moreover, the loss of mitochondrial membrane potential (MMP) is an early hallmark of apoptosis. Our findings indicate that SM treatment significantly reduced MMP, suggesting the involvement of mitochondrial dysfunction. Western blot analysis further revealed that SM treatment led to an upregulation of pro-apoptotic proteins Bax and cleaved caspase-3, alongside a downregulation of the anti-apoptotic protein Bcl-2, confirming that SM induces apoptosis in ICC cells via a mitochondrial-dependent pathway. Ferroptosis is a newly identified form of iron-dependent regulated cell death, characterized by excessive iron accumulation and lipid peroxidation. It is distinct from apoptosis but shares common regulatory networks. Growing evidence suggests that ferroptosis plays a critical role in tumorigenesis, cancer progression, and therapeutic responses^21^. However, whether ferroptosis contributes to SM-induced cytotoxicity in ICC cells remained unclear. In this study, we observed that SM treatment downregulated Slc7a11, Gpx4, and Nrf2 expression, key regulators of ferroptosis resistance. Additionally, SM significantly increased intracellular ROS production, Fe^2^⁺ levels, and lipid peroxidation by-products such as malondialdehyde (MDA), all of which are hallmarks of ferroptosis. Notably, pretreatment with ferroptosis inhibitor Fer-1 effectively rescued ICC cells from SM-induced cytotoxicity, confirming that ferroptosis is a key contributor to SM-mediated anti-ICC effects. Furthermore, transmission electron microscopy (TEM) revealed characteristic ferroptotic ultrastructural changes in SM-treated ICC cells. Collectively, these findings provide the direct evidence that SM inhibits ICC cell survival via a ferroptosis-dependent mechanism.

Although apoptosis and ferroptosis are distinct cell death mechanisms, accumulating evidence suggests a complex interplay between the two. Key regulators such as p53 and Nrf2 have been implicated in orchestrating the crosstalk between ferroptosis and apoptosis^22^. This interplay extends to multiple intracellular organelles, including mitochondria, lysosomes, and the endoplasmic reticulum, which coordinate stress responses and cell fate decisions^23^. Our findings align with previous studies, demonstrating that SM treatment induces oxidative stress in ICC cells, downregulates Nrf2, disrupts mitochondrial function, and ultimately activates both ferroptotic and apoptotic. These results suggest that SM induces both apoptotic and ferroptotic cell death pathways, which may contribute to its antitumor activity in ICC cells.

Transcriptomic sequencing revealed that differentially expressed genes were significantly enriched in the MAPK signaling pathway. The ERK pathway primarily directs a program of proliferation and survival, while the JNK pathway can promote either proliferation or apoptosis. Conversely, the p38 MAPK pathway is activated upon cellular stress and often engages pathways that can block proliferation or promote apoptosis^24^. The p38 MAPK pathway was reported to be involved in regulating apoptosis and ferroptosis^25^. Recent studies have found that higher p38 MAPK expression correlates with the increased expression of ferroptosis markers (Slc7a11, Gpx4, Nrf2) and apoptotic markers (caspase-3, PARP)^26–28^. In the present study, SM induced a significant increase in p38 phosphorylation. In contrast, phosphorylation levels of ERK and JNK showed no significant changes under the same conditions, indicating a selective activation of the p38 MAPK pathway by SM. SB203580, an inhibitor of p38 MAPK phosphorylation, was used to confirm the role of p38 MAPK in SM-induced apoptosis and ferroptosis. The apoptosis and ferroptosis abilities of ICC cells were reduced upon inhibition of p38 MAPK phosphorylation. It is well documented that ROS regulate the expression of p38 MAPK^29^. Reactive oxygen species are known to activate p38 MAPK through redox-sensitive upstream kinases such as ASK1 and MKK3/6^30–32^. In the present study, increased ROS generation was observed after SM treatment. In addition, the ROS scavenger NAC strongly reversed SM-induced apoptosis and ferroptosis; Taken together, these findings suggest that SM-induced apoptosis and ferroptosis in ICC cells are associated with the ROS/p38 MAPK signaling axis. Although our findings indicate that ROS accumulation precedes p38 activation in SM-treated ICC cells, the precise molecular mechanism underlying this activation warrants further investigation.

Finally, by establishing both an orthotopic and a subcutaneous tumor model, we confirmed that SM significantly inhibited ICC tumor growth after 18 days of treatment. Consistent with the in vivo findings, Western blot analysis demonstrated that SM treatment led to increased p38 phosphorylation, accompanied by the upregulation of apoptosis- and ferroptosis-related proteins, highlighting the role of the p38 MAPK pathway in SM-induced cell death.These results indicate that SM induces apoptosis and ferroptosis by activating the p38 MAPK signaling pathway, thereby suppressing ICC progression. However, several limitations should be acknowledged. First, the sample size of the orthotopic tumor model was relatively small, as it was designed as a preliminary exploratory validation. Second, the effects of SM on drug-resistant ICC cells were not explored and warrant further investigation.

## Conclusion

**Fig. 9:**
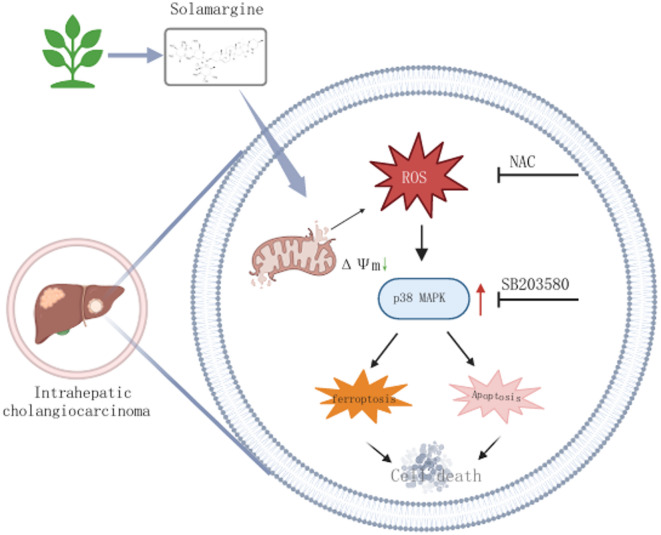
Graphical summary of the mechanism by which solamargine induces apoptosis and ferroptosis via the ROS/p38 MAPK signaling pathway in ICC.

In conclusion, SM inhibits ICC cell proliferation and is associated with the induction of apoptosis and ferroptosis, potentially involving the ROS/p38 MAPK signaling pathway(Fig. 9). SM exhibited potent anti-tumour activity in orthotopic tumor model and subcutaneous tumor model. The results of this study provide new insights into the potential efficacy of SM in the treatment of ICC.

## Supplementary Information

Supplementary Information 1.

Supplementary Information 2.

Supplementary Information 3.

## Data Availability

The RNA-seq datasets generated and/or analysed during the current study are available in the Gene Expression Omnibus (GEO) repository under accession number GSE308710. (https://www.ncbi.nlm.nih.gov/geo/query/acc.cgi?acc=GSE308710).

## Abbreviations

ANOVA: Analysis of variance
BTC: Biliary tract cancer
CCA: Cholangiocarcinoma
CQ: Chloroquine
DEGs: Differentially expressed gene
DMSO: Dimethylsulfoxide
ECL: Enhanced chemiluminescence
FBS: Fetal bovine serum
Fe^2+^: Ferrous iron
Fer-1: Ferrostatin-1
GEM: Gemcitabine
GSEA: Gene set enrichment analysis
GSH: Reduced glutathione
H&E: Hematoxylin–eosin staining
HIBEC: Human normal intrahepatic biliary epithelial cells
IC_50_: Half maximal inhibitory concentration
ICC: Intrahepatic cholangiocarcinoma
IHC: Immunohistochemistry
KEGG: Kyoto encyclopedia of genes and genomes
MAPK: Mitogen-activated protein kinases
MDA: Malondialdehyde
MMP: Mitochondrial membrane potential
mOS: Median overall survival
NAC: Acetylcysteine
Nec-1: Necrostatin-1
OS: Overall survival
PBS: Phosphate buffered saline
PCD: Programmed cell death
PI: Propidium iodide
PVDF: Polyvinylidene Fluoride
ROS: Reactive oxygen species
SB203580: Adezmapimod
SD: Standard deviation
SDS- PAGE: Sodium dodecyl sulfate–polyacrylamide gel electrophoresis
SM: Solamargine
TEM: Transmission electron microscopy
TUNEL: Terminal deoxynucleotidyl transferase deoxyuridine triphosphate nick-end labeling
WB: Western blotting

## References

[1] Bray, F. et al. Global cancer statistics 2018: Globocan estimates of incidence and mortality worldwide for 36 cancers in 185 countries. *CA. Cancer. J. Clin.* **68**, 394–424 (2018). 30207593 10.3322/caac.21492

[2] Yao, K. J., Jabbour, S., Parekh, N., Lin, Y. & Moss, R. A. Increasing mortality in the United States from cholangiocarcinoma: An analysis of the National Center for Health Statistics Database. *BMC Gastroenterol.* **16**, 117 (2016). 27655244 10.1186/s12876-016-0527-z PMC5031355

[3] Zhang, H., Lv, J. L., Zheng, Q. S. & Li, J. Active components of *Solanum nigrum* and their antitumor effects: A literature review. *Front. Oncol.* **13**, 1329957 (2023). 38192621 10.3389/fonc.2023.1329957 PMC10773844

[4] Kalalinia, F. & Karimi-Sani, I. Anticancer properties of Solamargine: A systematic review. *Phytother. Res.* **31**, 858–870 (2017). 28383149 10.1002/ptr.5809

[5] Wei, S., Han, C., Mo, S., Huang, H. & Luo, X. Advancements in programmed cell death research in antitumor therapy: A comprehensive overview. *Apoptosis* **30**, 401–421 (2025). 39487314 10.1007/s10495-024-02038-0

[6] Jalali-Zefrei, F., Mousavi, S. M., Delpasand, K., Shourmij, M. & Farzipour, S. Role of non-coding RNAs on the radiotherapy sensitivity and resistance in cancer cells. *Curr. Gene Ther.* **25**, 113–135 (2025). 38676526 10.2174/0115665232301727240422092311

[7] Xie, Y., Hou, W., Song, X. et al. Ferroptosis: Process and Function **23** 369–79 (2016). 10.1038/cdd.2015.158 PMC5072448 26794443

[8] Zhong, H., Liu, H. & Fu, Q. Ferroptosis as a therapeutic target in neurodegenerative diseases: Exploring the mechanisms and potential of treating Alzheimer’s Disease and Parkinson’s Disease. *Protein. Pept. Lett.* **31**, 759–772 (2024). 39513303 10.2174/0109298665333926240927074528

[9] Zhao, X., Zhang, M., He, J., Li, X. & Zhuang, X. Emerging insights into ferroptosis in cholangiocarcinoma (Review). *Oncol. Lett.* **28**, 606 (2024). 39483963 10.3892/ol.2024.14739 PMC11526429

[10] Lee, Y. S. et al. Bax-dependent mitochondrial pathway mediates the crosstalk between ferroptosis and apoptosis. *Apoptosis* **25**, 625–631 (2020). 32737652 10.1007/s10495-020-01627-z PMC7529973

[11] Wang, X., Tan, X., Zhang, J., Wu, J. & Shi, H. The emerging roles of MAPK-AMPK in ferroptosis regulatory network. *Cell. Commun. Signal.* **21**, 200 (2023). 37580745 10.1186/s12964-023-01170-9 PMC10424420

[12] Li, J., Gao, H., Wang, P. et al. Plumbagin induces G2/M arrest and apoptosis and ferroptosis Via Ros/P38 Mapk pathway in human osteosarcoma cells. **103** 222–36 (2024).

[13] Su, L. J. et al. Reactive oxygen species-induced lipid peroxidation in apoptosis, autophagy, and ferroptosis. *Oxid. Med. Cell. Longev.* **2019**, 5080843 (2019). 31737171 10.1155/2019/5080843 PMC6815535

[14] Lyu, F. et al. Omicstudio: A composable bioinformatics cloud platform with real-time feedback that can generate high-quality graphs for publication. *iMeta* **2**, e85 (2023). 38868333 10.1002/imt2.85 PMC10989813

[15] Pfeffer, C. M. & Singh Apoptosis: A Target for Anticancer Therapy *J. Int. J. Mol. ences Atk.* **19** 448 (2018). 10.3390/ijms19020448 PMC5855670 29393886

[16] Newton, K., Strasser, A., Kayagaki, N. & Vishva, M. Cell Death. *J. Cell Dixit.* **187** 235–56 (2024). 10.1016/j.cell.2023.11.044 38242081

[17] Tsung, C., Quinn, P.L. & Aslam Management of intrahepatic cholangiocarcinoma: a narrative review J. Cancers Ejaz **16** 739 (2024). 10.3390/cancers16040739 PMC10886475 38398130

[18] Pei, H. et al. *Solanum nigrum* Linn.: Advances in anti-cancer activity and mechanism in digestive system tumors. *Med. Oncol.* **40**, 311 (2023). 37775552 10.1007/s12032-023-02167-7

[19] Yin, S. et al. Solamargine induces hepatocellular carcinoma cell apoptosis and autophagy via inhibiting Lif/Mir-192-5p/Cyr61/Akt signaling pathways and eliciting immunostimulatory tumor microenvironment. *J. Hematol. Oncol.* **15**, 32 (2022). 35313929 10.1186/s13045-022-01248-w PMC8935708

[20] Han, Q. et al. Solamargine induces autophagy-mediated apoptosis and enhances Bortezomib activity in multiple myeloma. *Clin. Exp. Pharmacol. Physiol.* **49**, 674–685 (2022). 35294057 10.1111/1440-1681.13643 PMC9310729

[21] Zhou, Q. et al. Ferroptosis in cancer: From molecular mechanisms to therapeutic strategies. *Signal Transduct. Target. Ther.* **9**, 55 (2024). 38453898 10.1038/s41392-024-01769-5 PMC10920854

[22] Vinik, Y. & Lev, S. A snapshot into the transcriptomic landscape of apoptosis and ferroptosis in cancer. *Cell Death Dis.* **15**, 371 (2024). 38811541 10.1038/s41419-024-06766-8 PMC11137012

[23] Wu, P., Zhang, X., Duan, D. & Zhao, L. Organelle-Specific Mechanisms in Crosstalk between Apoptosis and Ferroptosis. *Oxid. Med. Cell. Longev.* **2023**, 3400147 (2023). 36644574 10.1155/2023/3400147 PMC9836800

[24] Kennedy, N.J., Cellurale, C. & Roger, J. A Radical Role for P38 Mapk in Tumor Initiation. *J. Cancer cell Davis.* **11** 101–03 (2007). 10.1016/j.ccr.2007.01.009 17292820

[25] Chang, W. T. et al. A Marine Terpenoid, Heteronemin, Induces Both the Apoptosis and Ferroptosis of Hepatocellular Carcinoma Cells and Involves the Ros and Mapk Pathways. *Oxid. Med. Cell. Longev.* **2021**, 7689045 (2021). 33488943 10.1155/2021/7689045 PMC7803406

[26] Wang, H. H., Fan, S. Q., Zhan, Y. T., Peng, S. P. & Wang, W. Y. Suppression of the Slc7a11/Glutathione axis causes ferroptosis and apoptosis and alters the mitogen-activated protein kinase pathway in nasopharyngeal carcinoma. *Int. J. Biol. Macromol.* **254**, 127976 (2024). 37951442 10.1016/j.ijbiomac.2023.127976

[27] Zhu, J. et al. Escin induces caspase-dependent apoptosis and autophagy through the ros/P38 mapk signalling pathway in human osteosarcoma cells in vitro and in vivo. *Cell Death Dis.* **8**, e3113 (2017). 29022891 10.1038/cddis.2017.488 PMC5682655

[28] Guo, F. et al. Juglone induces ferroptosis in glioblastoma cells by inhibiting the Nrf2-Gpx4 axis through the phosphorylation of P38mapk. *Chin. Med.* **19**, 52 (2024). 38520025 10.1186/s13020-024-00920-2 PMC10958923

[29] Son, Y., Cheong, Y.K. Kim, N.H., Chung, H. T., Kang, D.G. & Hyun-Ock Mitogen activated protein kinases and reactive oxygen species: how can ros activate mapk pathways?. *J. Signal. Transduct. Pae* **2011** 792639 (2011). 10.1155/2011/792639 PMC3100083 21637379

[30] Zarubin, T. & Han, J. Activation and signaling of the P38 map kinase pathway. *Cell Res.* **15**, 11–18 (2005). 15686620 10.1038/sj.cr.7290257

[31] García-Hernández, L. et al. The P38 MAPK components and modulators as biomarkers and molecular targets in *cancer. Int. J. Mol. Sci.* **23**, 370 (2021). 35008796 10.3390/ijms23010370 PMC8745478

[32] Kennedy, N. J., Cellurale, C. & Davis, R. J. A radical role for P38 Mapk in tumor initiation. *Cancer Cell* **11**, 101–103 (2007). 17292820 10.1016/j.ccr.2007.01.009

