# Supplementary material for: Solamargine induces apoptosis and ferroptosis through the ROS/p38 MAPK signalling pathway in intrahepatic cholangiocarcinoma
Source: Sci Rep. 2026 Apr 24;16:19045. doi: 10.1038/s41598-026-49458-3 (PMC13280501; doi:10.1038/s41598-026-49458-3)

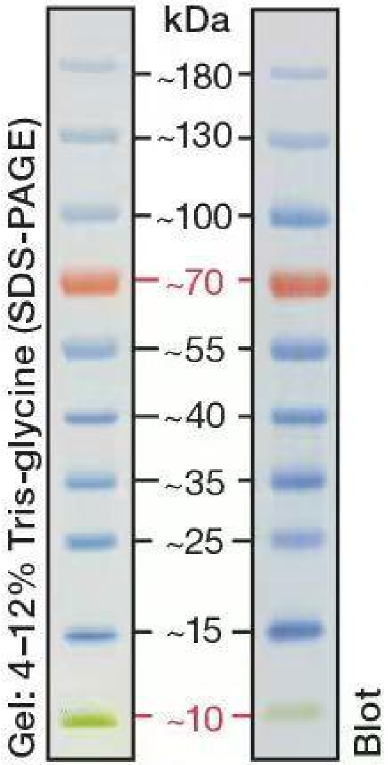
kDa
~180
~130
~100
~70
~55
~40
~35
~25
~15
~10
Gel: 4–12% Tris-glycine (SDS-PAGE)
Blot

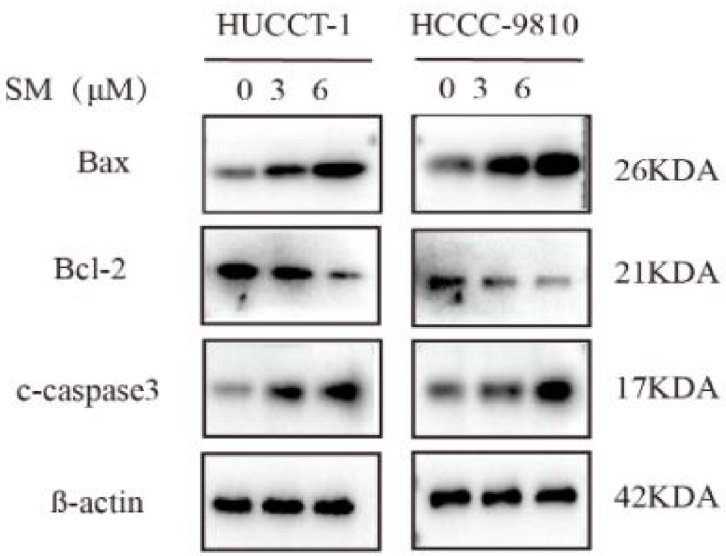
HUCCT-1
HCCC-9810
SM（μM）
0 3 6
0 3 6
Bax
Bcl-2
c-caspase3
ß-actin
26KDA
21KDA
17KDA
42KDA

figure 2E

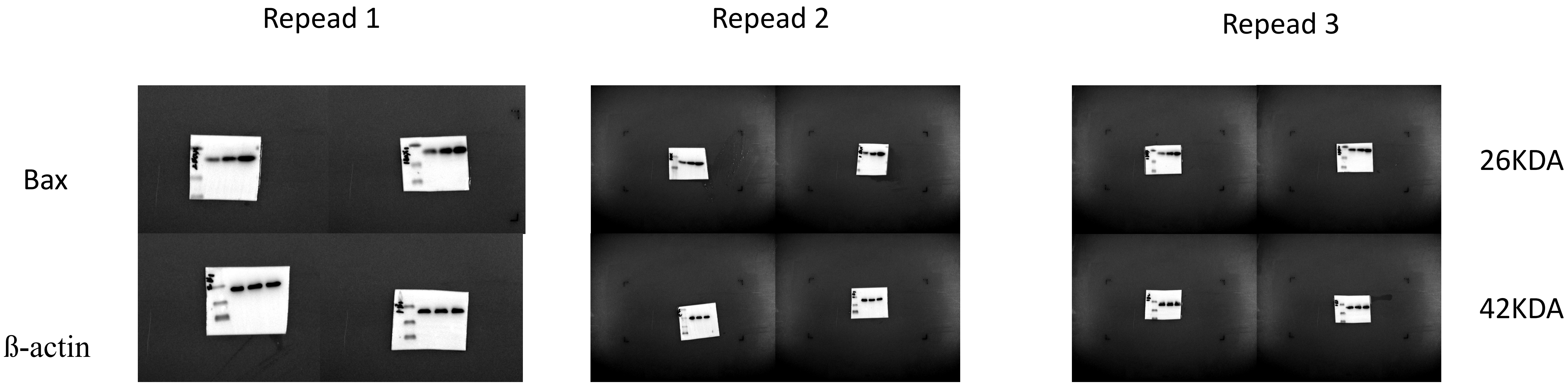
Repead 1
Repead 2
Repead 3
Bax
ß-actin
26KDA
42KDA

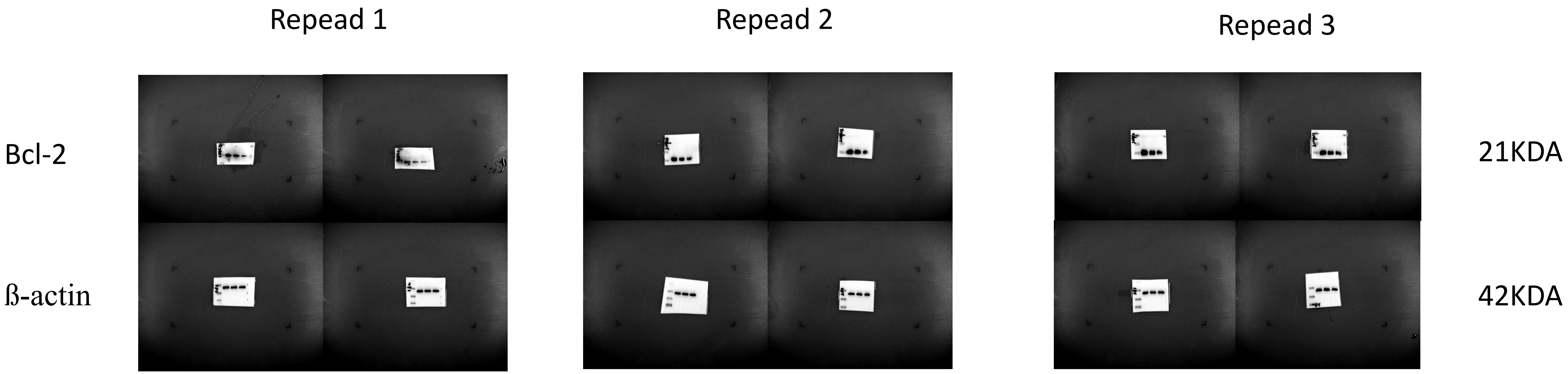

Repead 1
Repead 2
Repead 3
Bcl-2
ß-actin
21KDA
42KDA

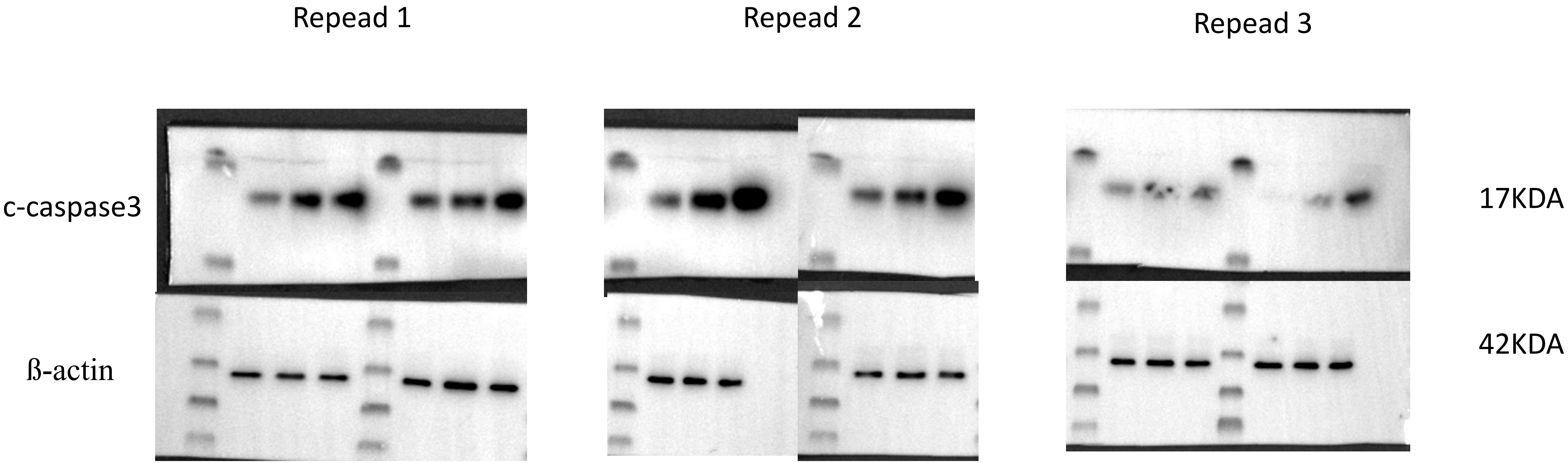
Repead 1
Repead 2
Repead 3
c-caspase3
ß-actin
17KDA
42KDA

figure 5E

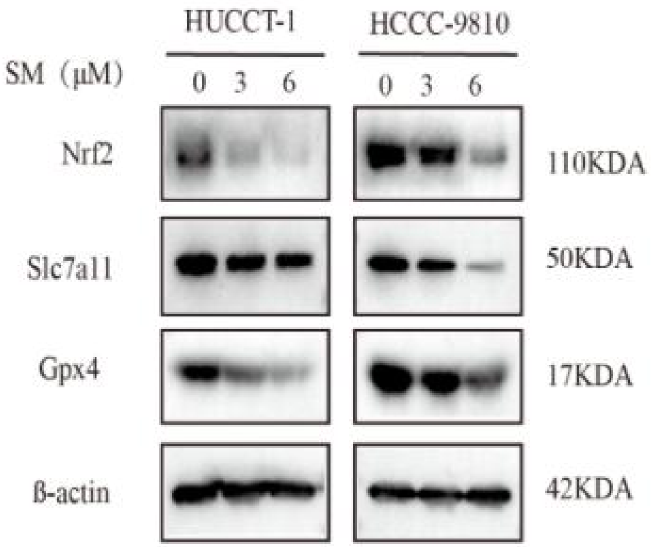

HUCCT-1
HCCC-9810
SM（μM）
0 3 6
0 3 6
Nrf2
Slc7a11
Gpx4
ß-actin
110KDA
50KDA
17KDA
42KDA

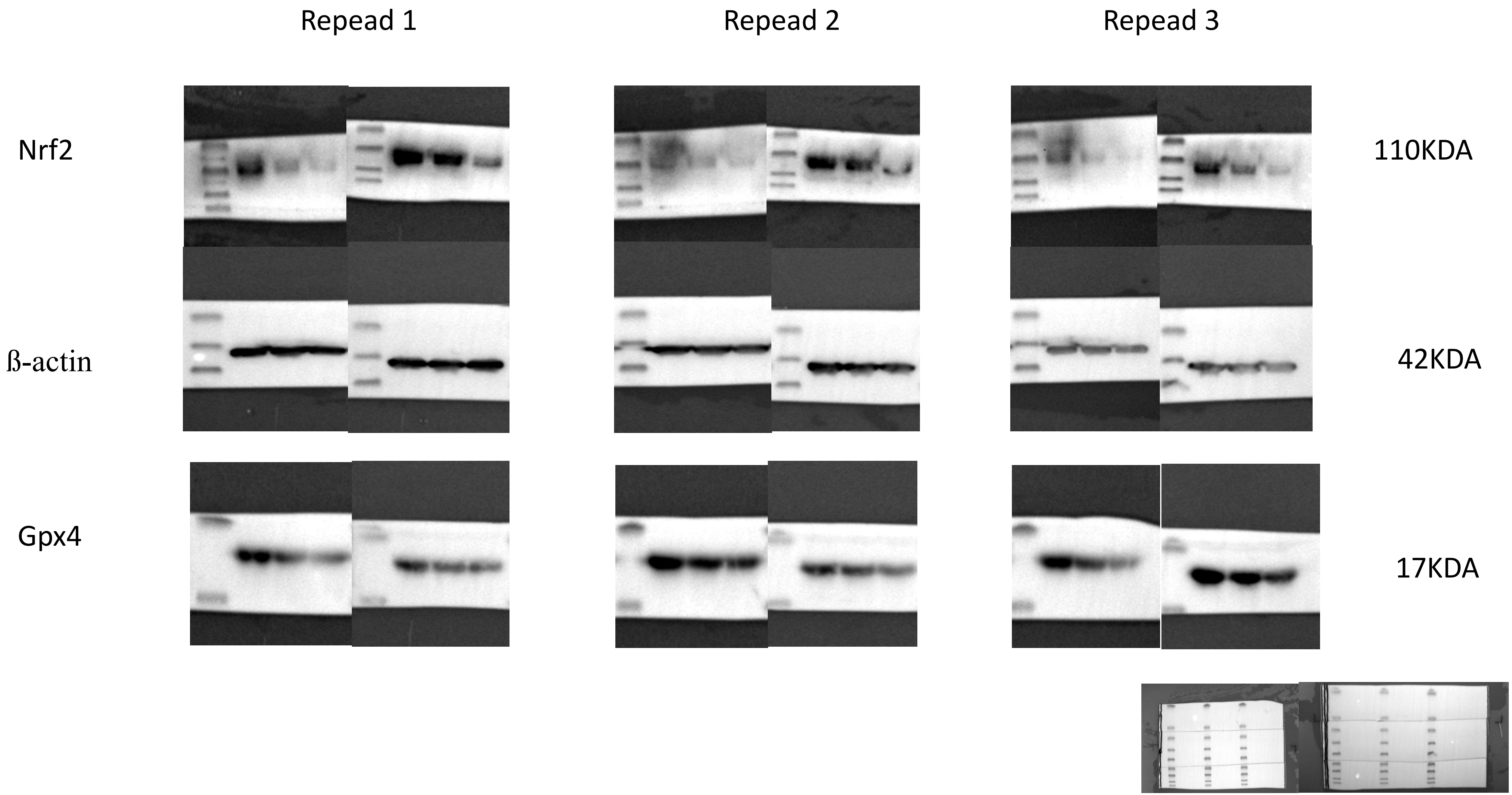
Repead 1
Repead 2
Repead 3
Nrf2
110KDA
ß-actin
42KDA
Gpx4
17KDA

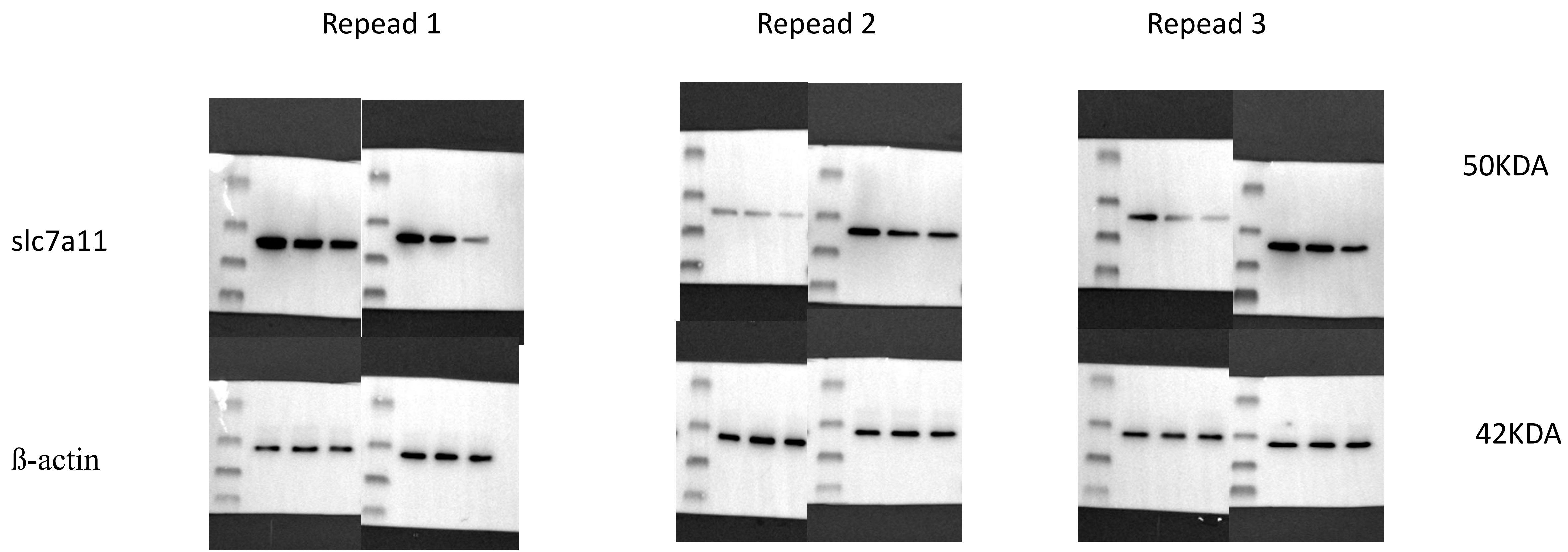
Repead 1
Repead 2
Repead 3
slc7a11
ß-actin
50KDA
42KDA

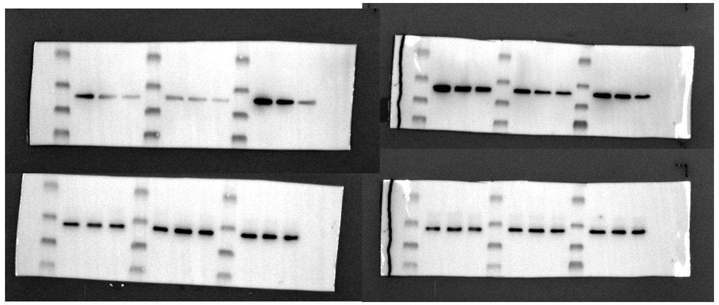

figure 6A

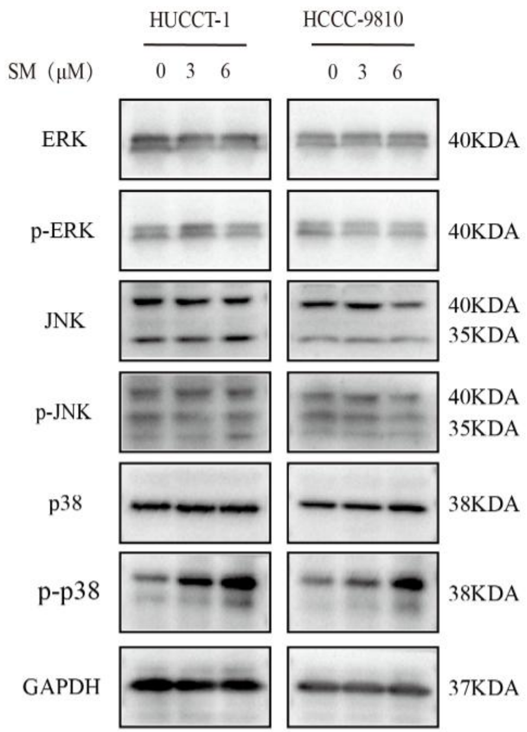
HUCCT-1
HCCC-9810
SM (μM)
0 3 6
0 3 6
ERK
p-ERK
JNK
p-JNK
p38
p-p38
GAPDH
40KDA
40KDA
40KDA
35KDA
40KDA
35KDA
38KDA
38KDA
37KDA

ERK

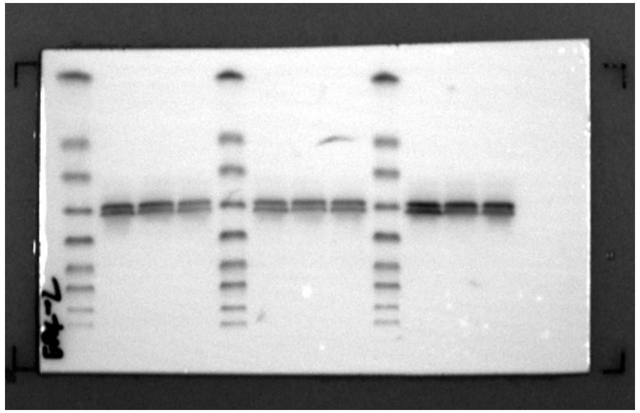

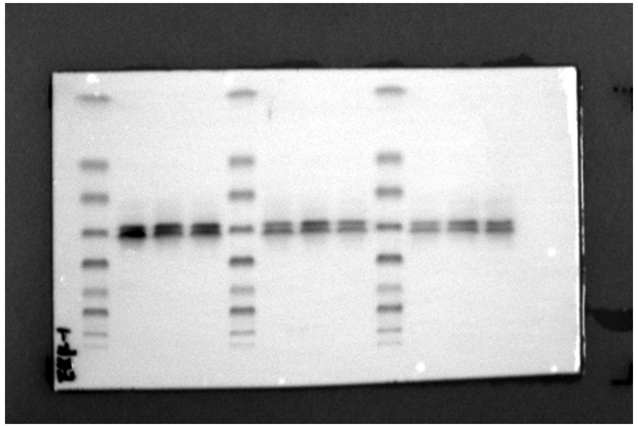

40KDA

P-ERK

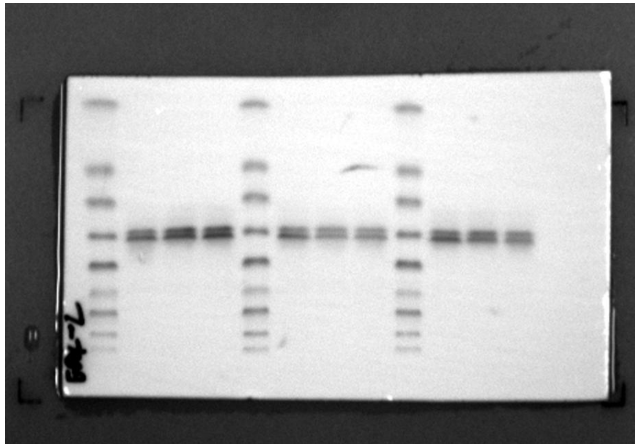

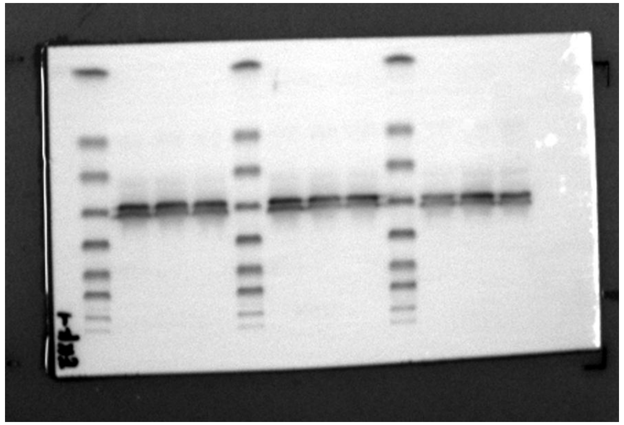

40KDA

GAPDH

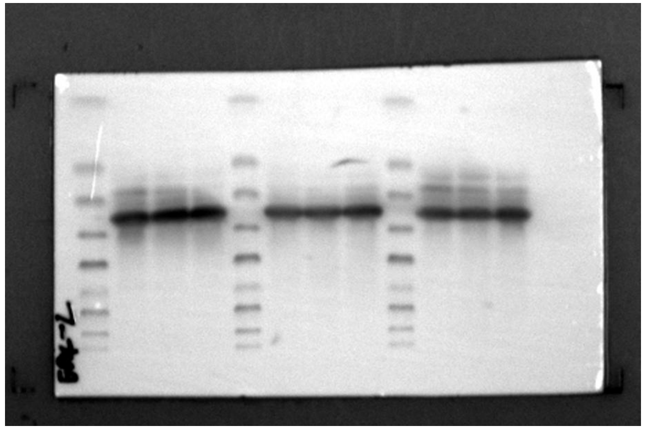

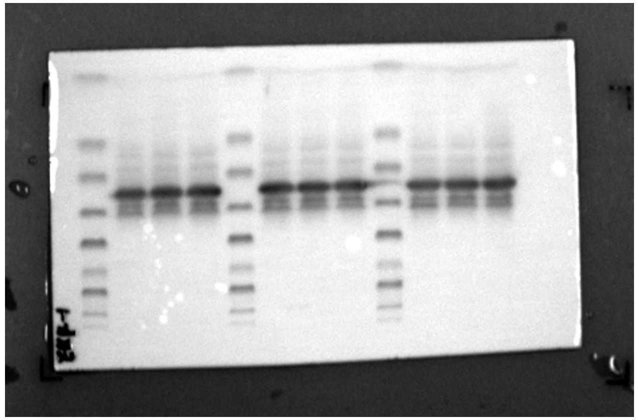

37KDA

JNK

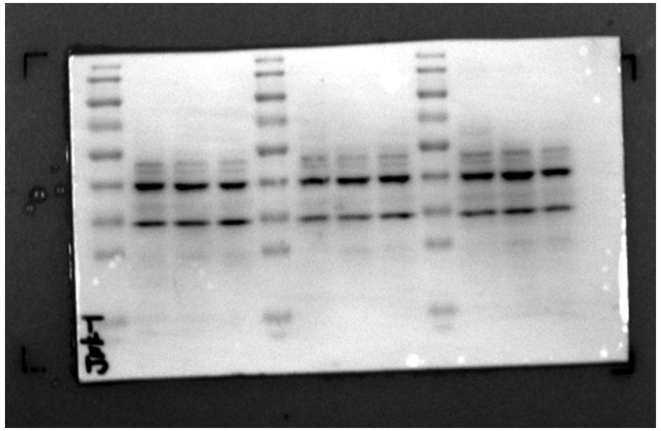

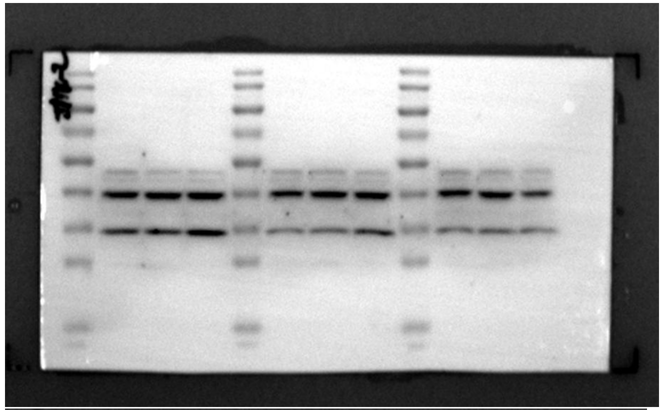

40KDA

35KDA

P-JNK

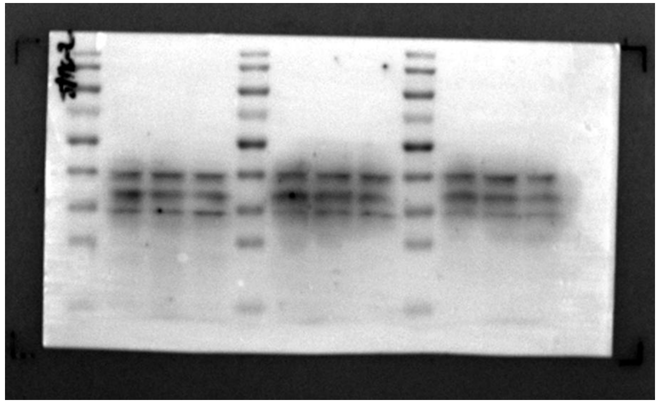

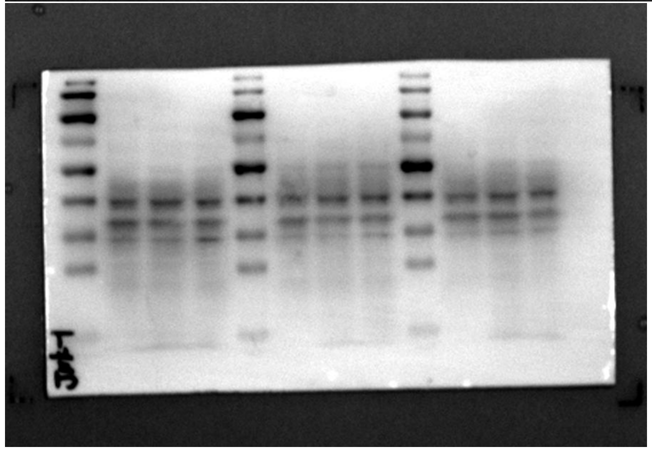

40KDA

35KDA

GAPDH

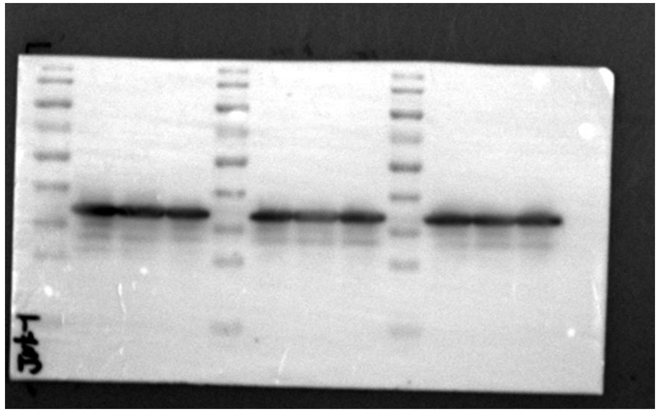

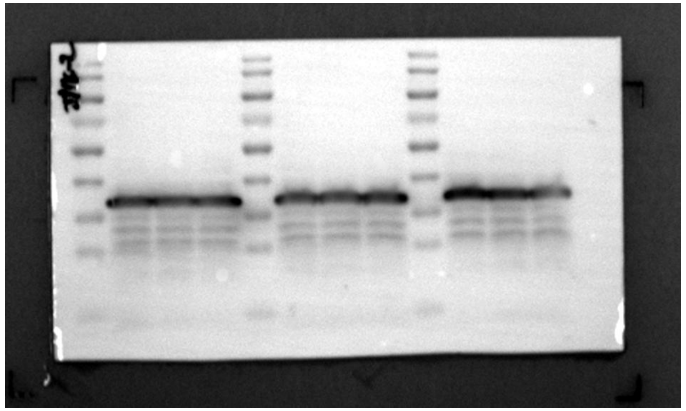

37KDA

P38

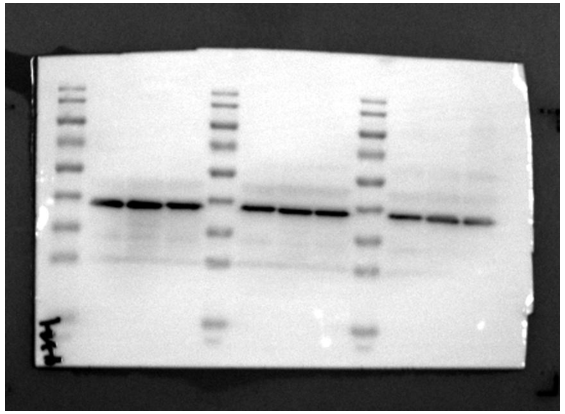

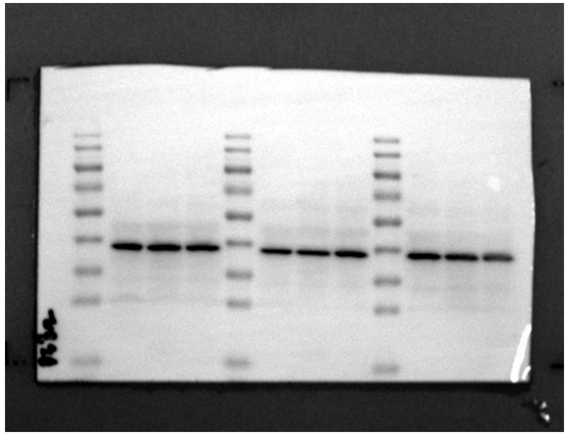

38KDA

P-P38

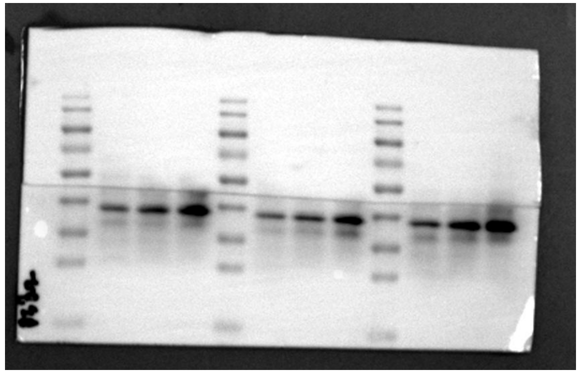

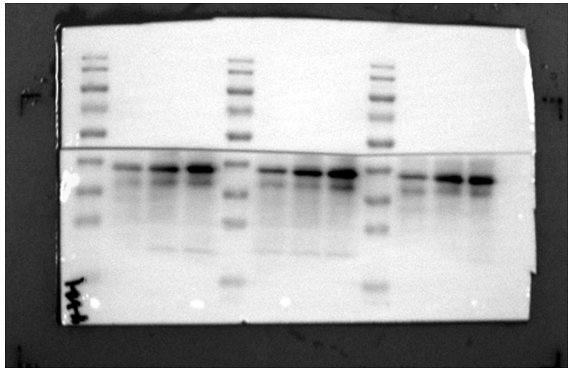

38KDA

GAPDH

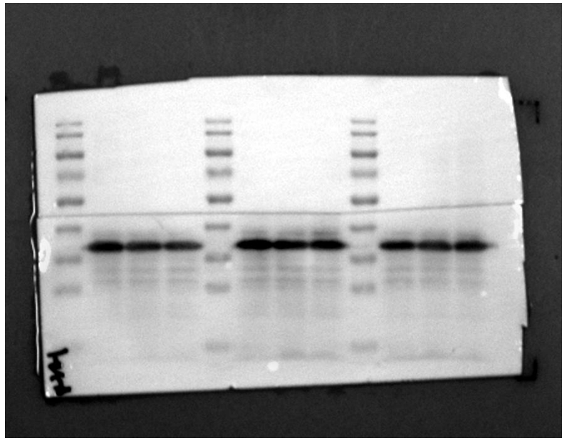

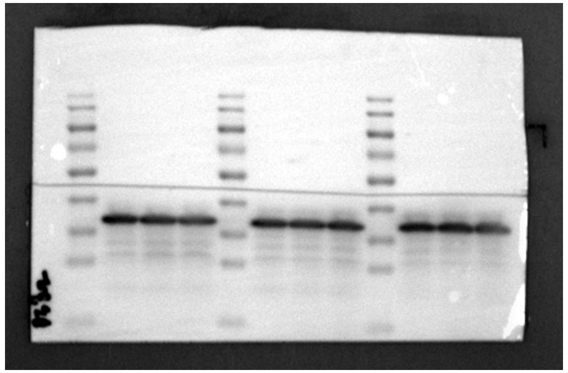

37KDA

figure 6B

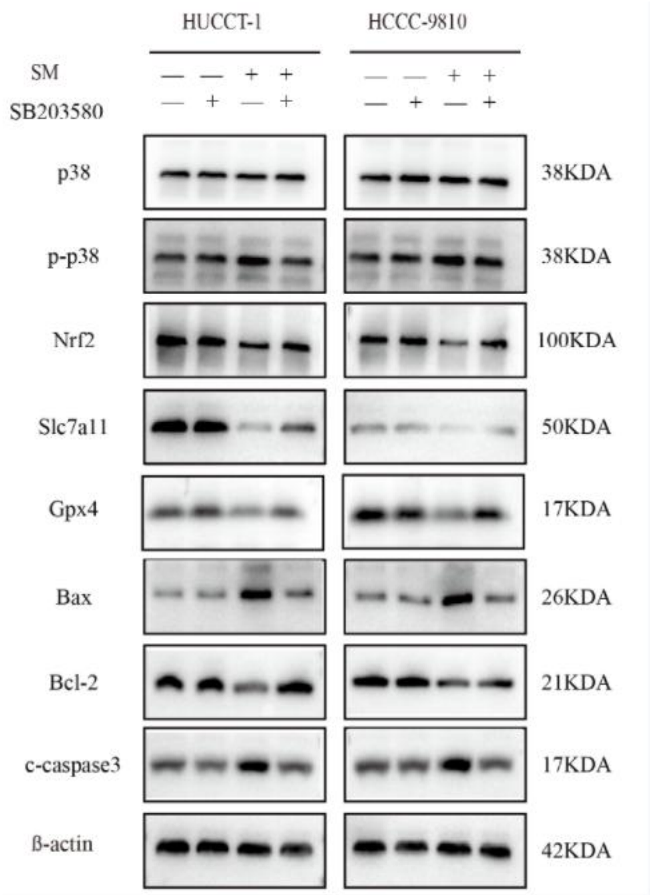
HUCCT-1
HCCC-9810
SM
SB203580
p38
p-p38
Nrf2
Slc7a11
Gpx4
Bax
Bcl-2
c-caspase3
ß-actin
38KDA
38KDA
100KDA
50KDA
17KDA
26KDA
21KDA
17KDA
42KDA

Repead 1

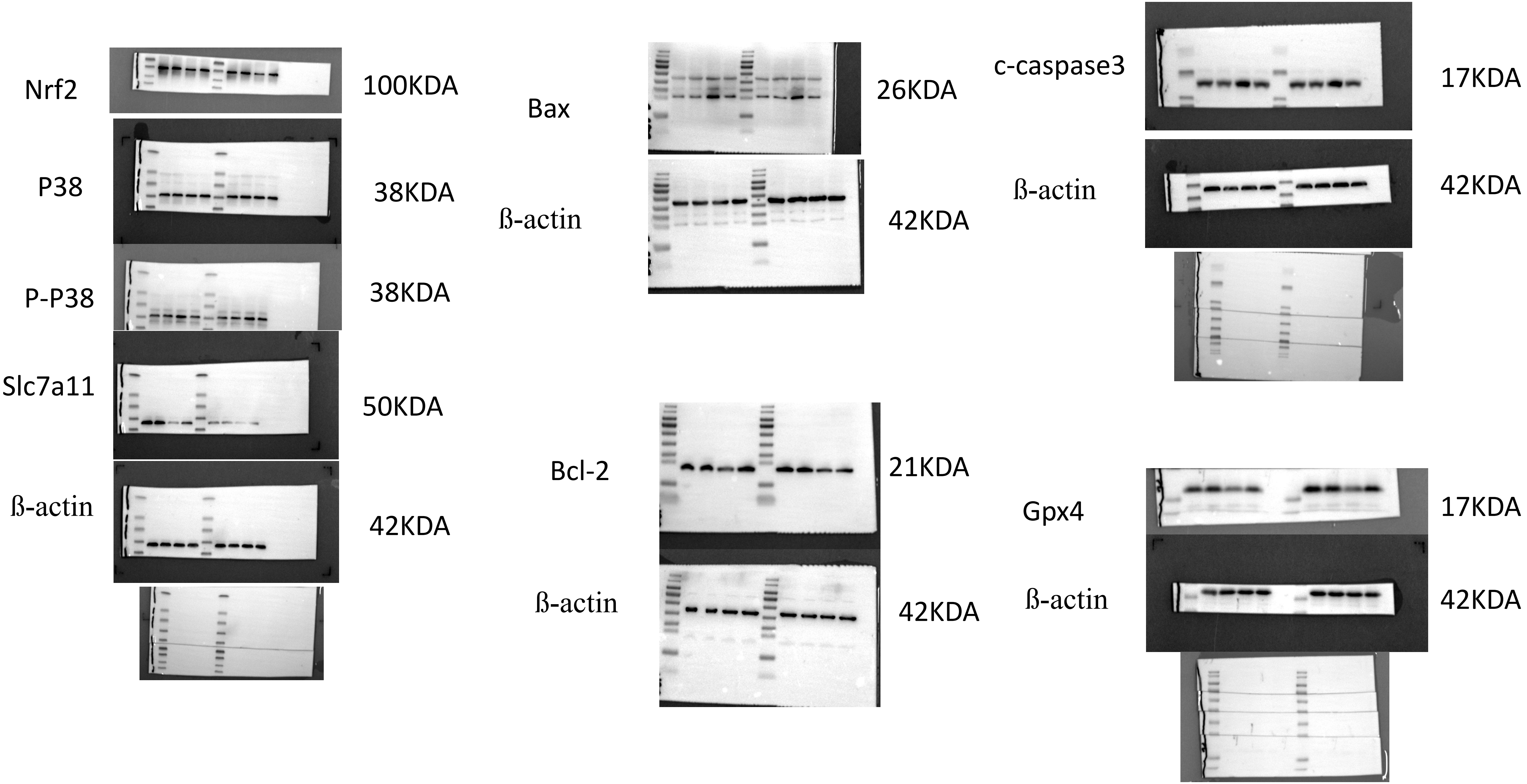
Nrf2
100KDA
P38
38KDA
P-P38
38KDA
Slc7a11
50KDA
ß-actin
42KDA
Bax
26KDA
ß-actin
42KDA
Bcl-2
21KDA
ß-actin
42KDA
c-caspase3
17KDA
ß-actin
42KDA
Gpx4
17KDA
ß-actin
42KDA

Repead 2

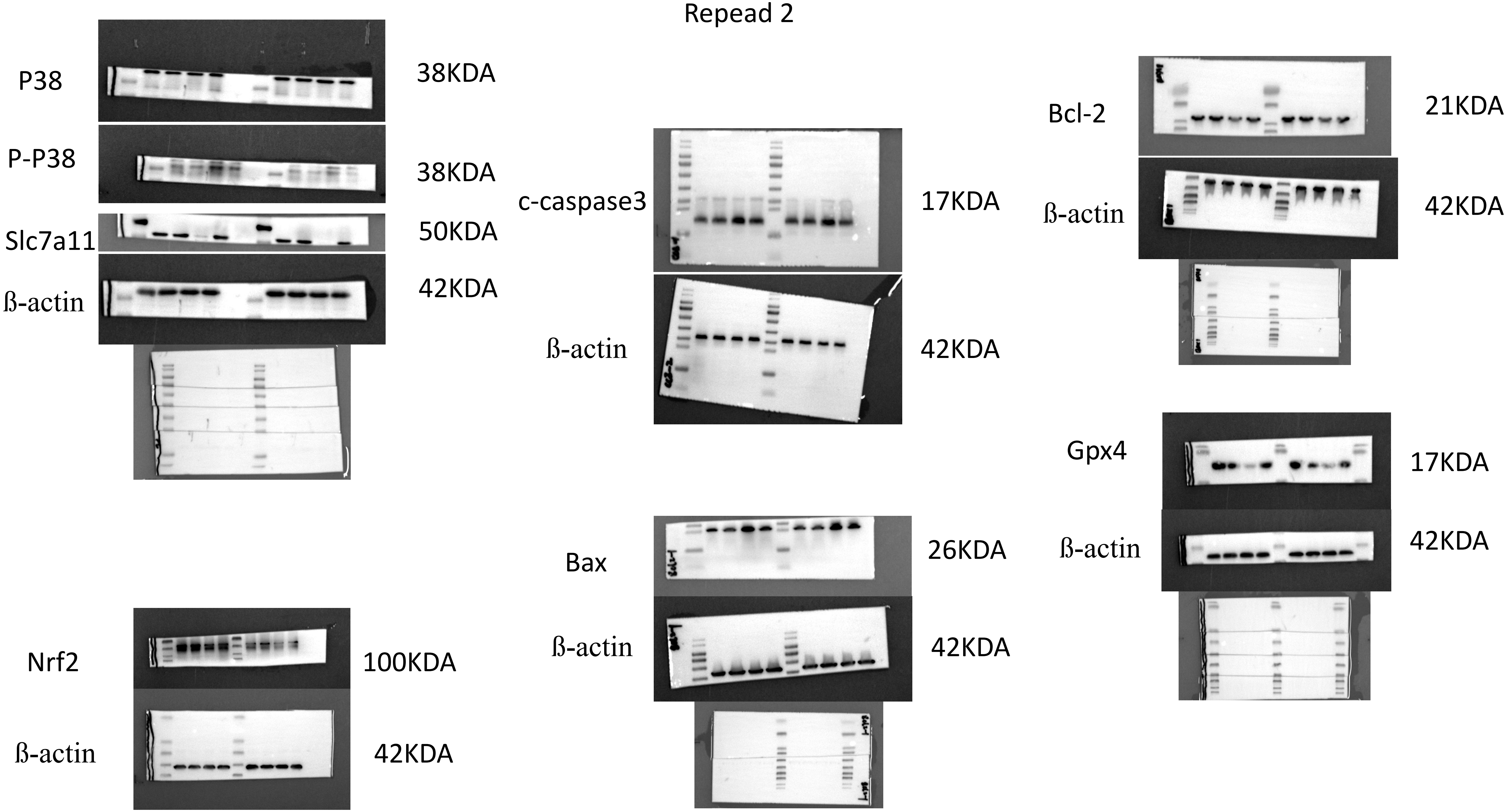
P38
38KDA
P-P38
38KDA
Slc7a11
50KDA
ß-actin
42KDA
c-caspase3
17KDA
ß-actin
42KDA
Bcl-2
21KDA
ß-actin
42KDA
Gpx4
17KDA
ß-actin
42KDA
Bax
26KDA
ß-actin
42KDA
Nrf2
100KDA
ß-actin
42KDA

Repead3

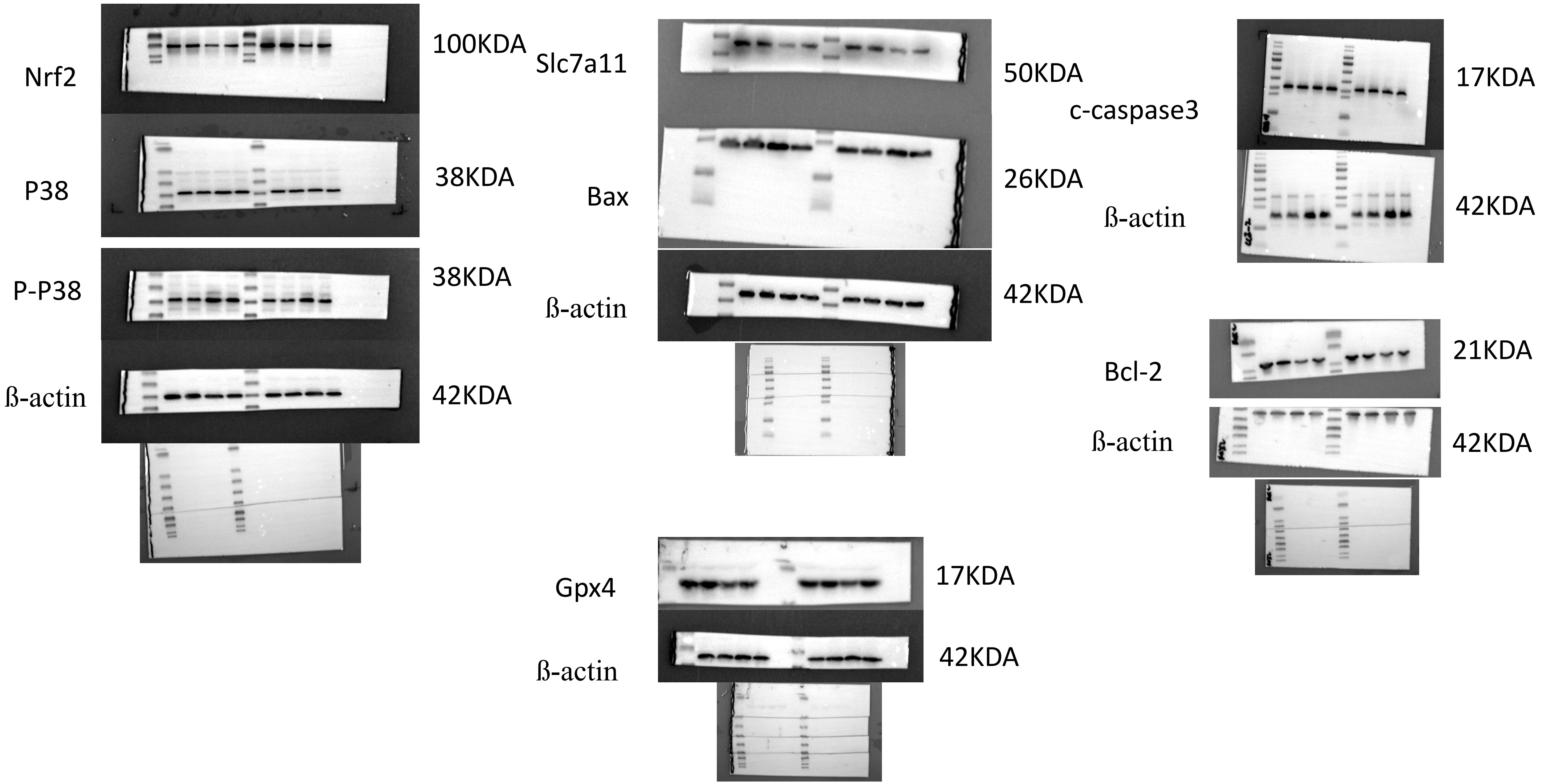
Nrf2
100KDA
P38
38KDA
P-P38
38KDA
ß-actin
42KDA
Slc7a11
50KDA
Bax
26KDA
ß-actin
42KDA
Gpx4
17KDA
ß-actin
42KDA
c-caspase3
17KDA
ß-actin
42KDA
Bcl-2
21KDA
ß-actin
42KDA

figure7C

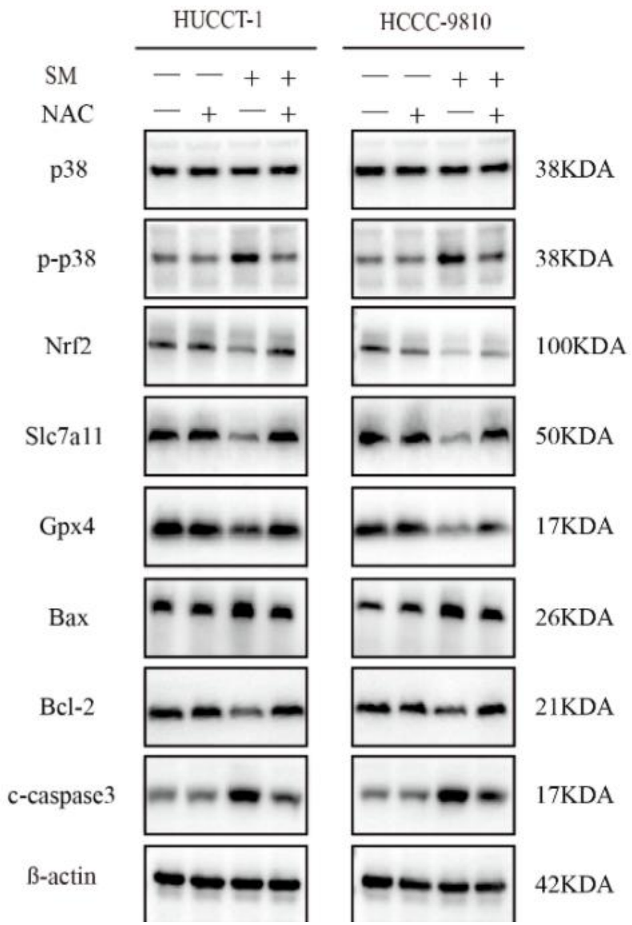

HUCCT-1
HCCC-9810
SM
NAC
p38
p-p38
Nrf2
Slc7a11
Gpx4
Bax
Bcl-2
c-caspase3
ß-actin
38KDA
38KDA
100KDA
50KDA
17KDA
26KDA
21KDA
17KDA
42KDA

Repead1

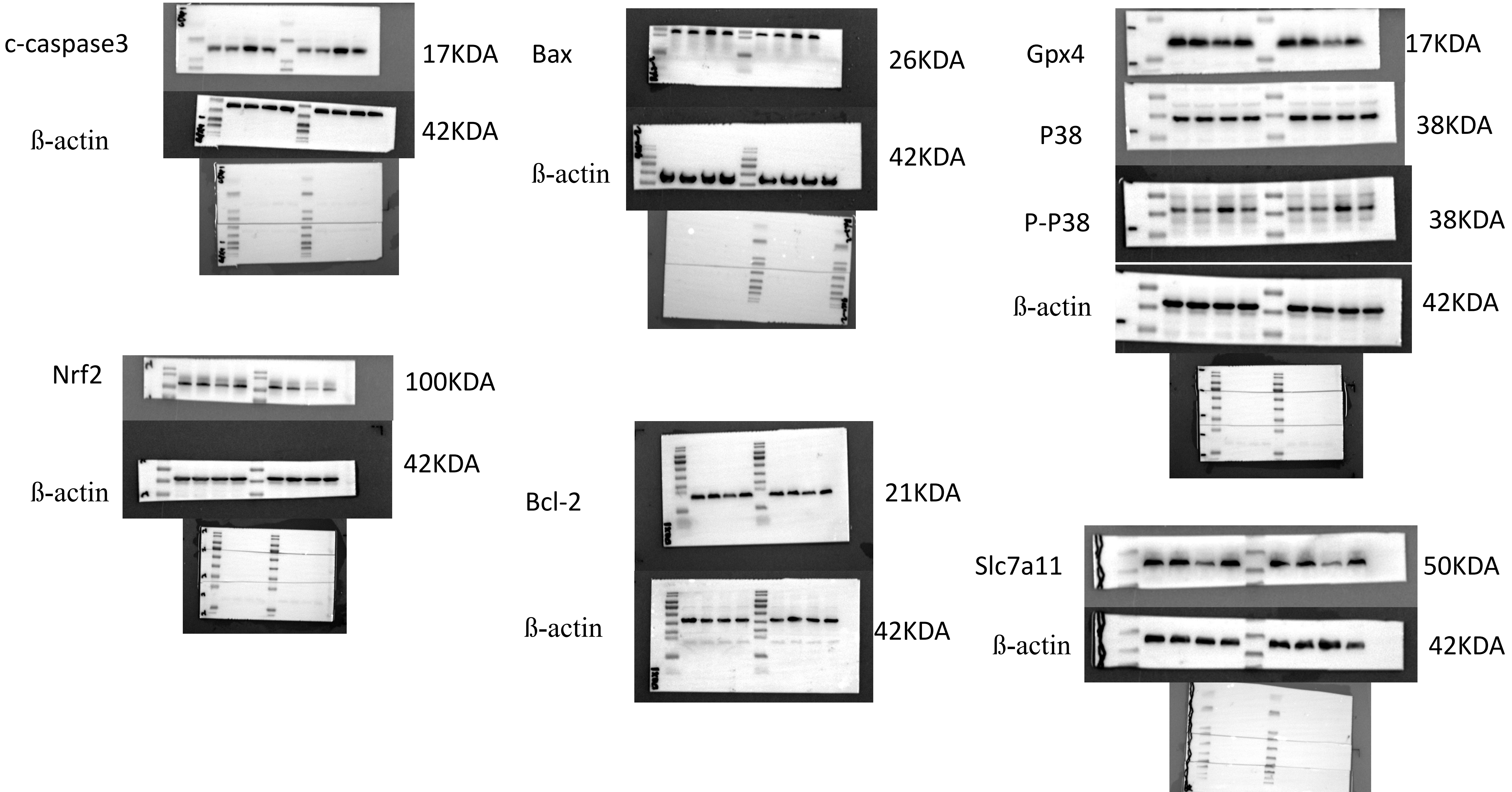
c-caspase3
17KDA
ß-actin
42KDA
Bax
26KDA
ß-actin
42KDA
Gpx4
17KDA
P38
38KDA
P-P38
38KDA
ß-actin
42KDA
Nrf2
100KDA
ß-actin
42KDA
Bcl-2
21KDA
ß-actin
42KDA
Slc7a11
50KDA
ß-actin
42KDA

Repead2

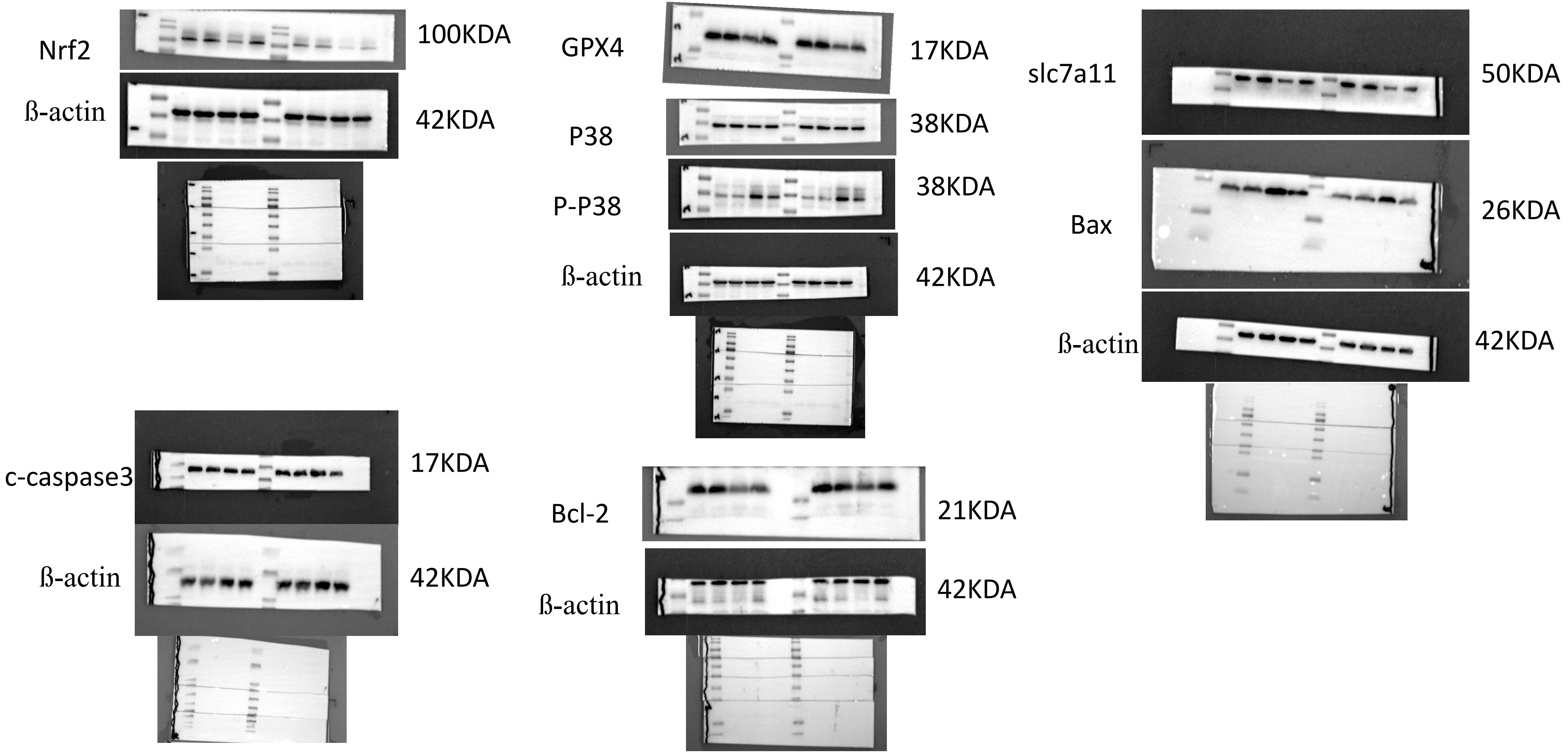

Nrf2
100KDA
ß-actin
42KDA
GPX4
17KDA
P38
38KDA
P-P38
38KDA
ß-actin
42KDA
slc7a11
50KDA
Bax
26KDA
ß-actin
42KDA
c-caspase3
17KDA
ß-actin
42KDA
Bcl-2
21KDA
ß-actin
42KDA

Repead3

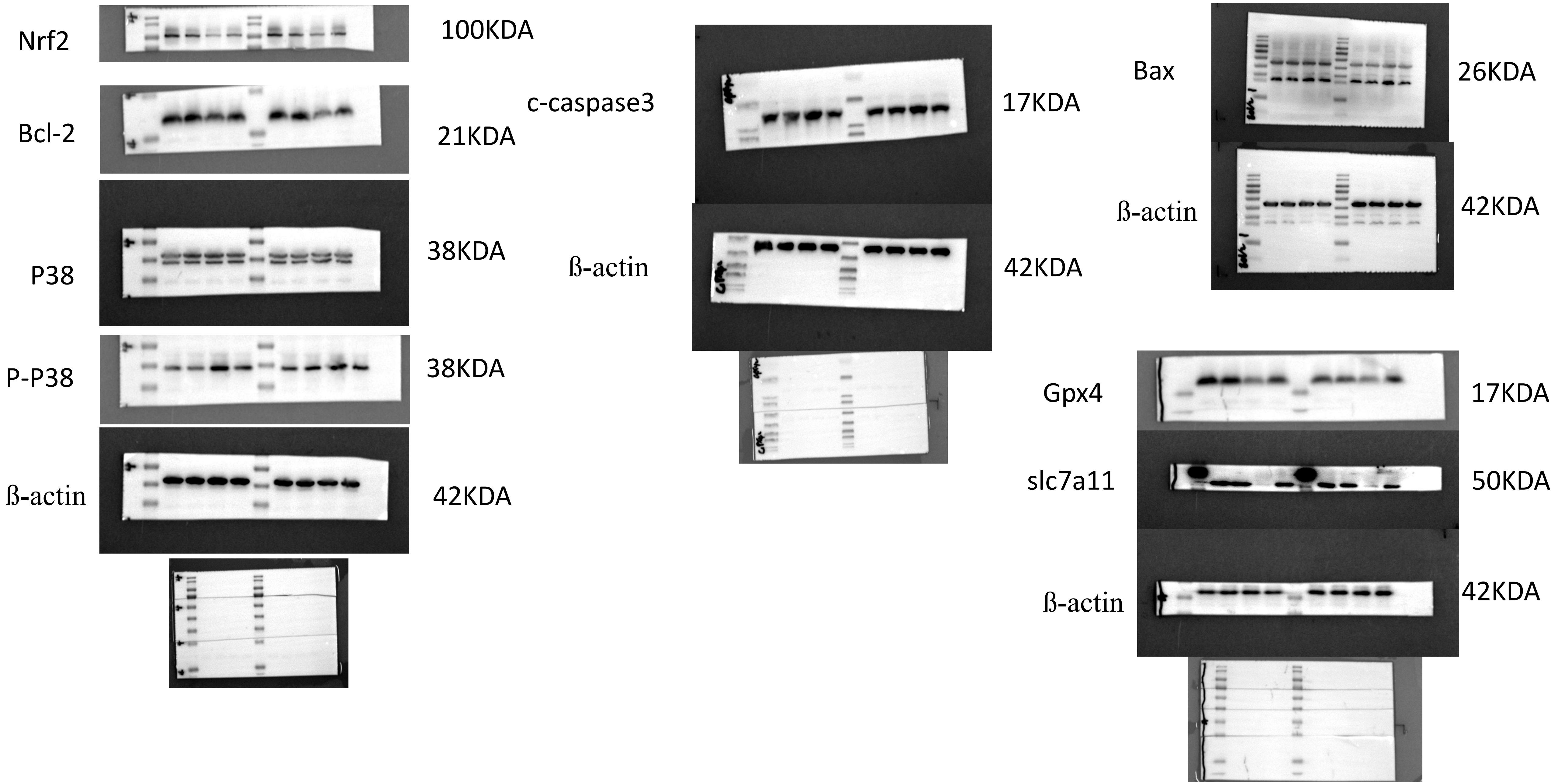
Nrf2 100KDA
Bcl-2 21KDA
P38 38KDA
P-P38 38KDA
ß-actin 42KDA
c-caspase3 17KDA
ß-actin 42KDA
Bax 26KDA
ß-actin 42KDA
Gpx4 17KDA
slc7a11 50KDA
ß-actin 42KDA

figure 8I

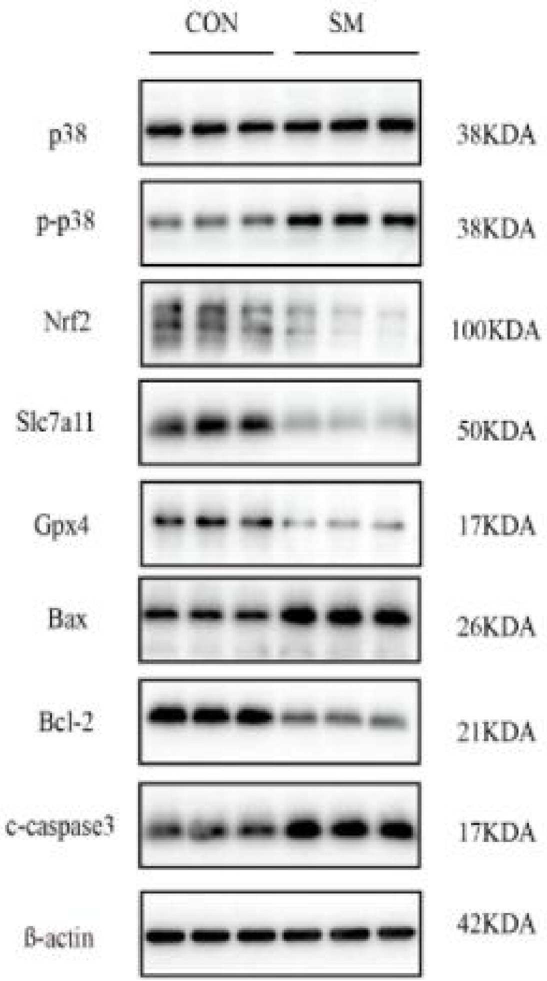

CON
SM
p38
38KDA
p-p38
38KDA
Nrf2
100KDA
Slc7a11
50KDA
Gpx4
17KDA
Bax
26KDA
Bcl-2
21KDA
c-caspase3
17KDA
ß-actin
42KDA

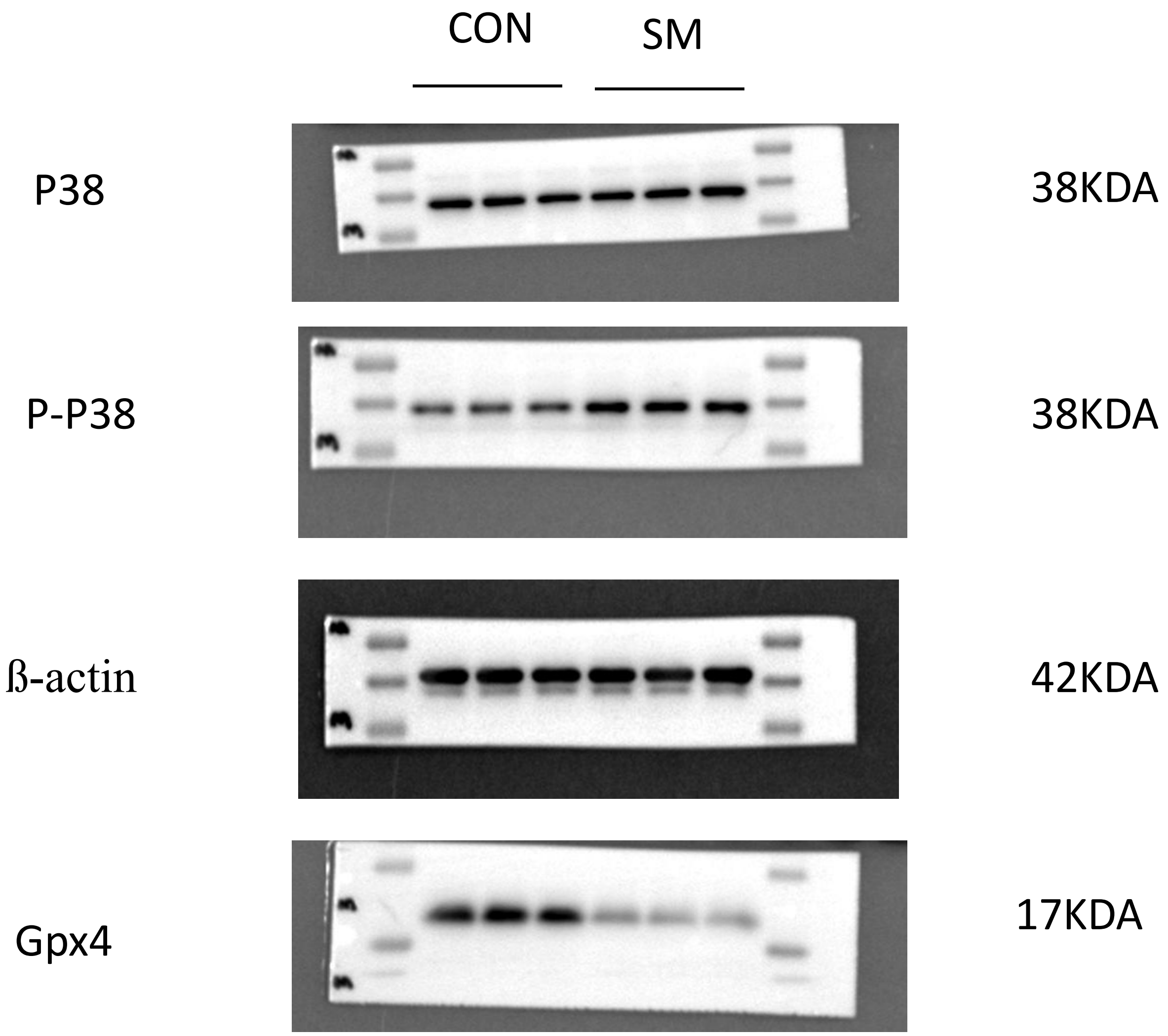
CON
SM
P38
38KDA
P-P38
38KDA
ß-actin
42KDA
Gpx4
17KDA

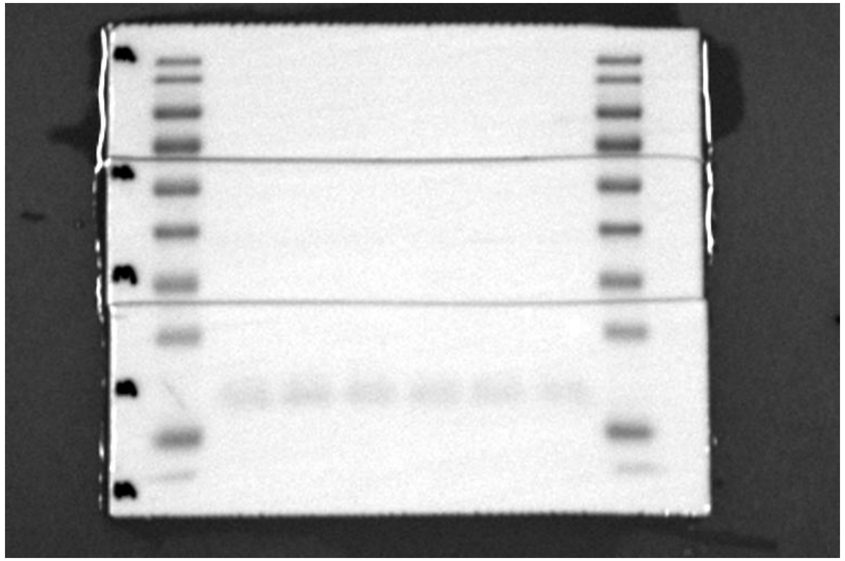

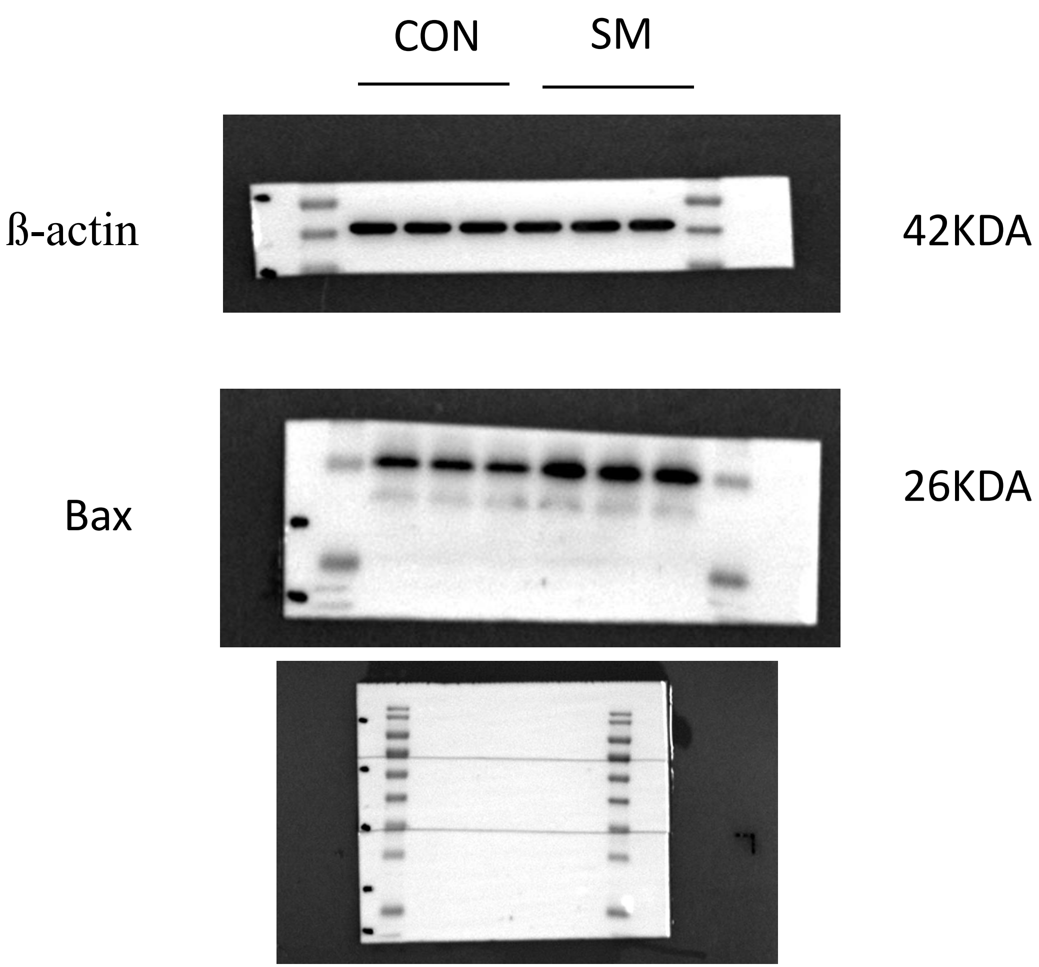
CON
SM
ß-actin
42KDA
Bax
26KDA

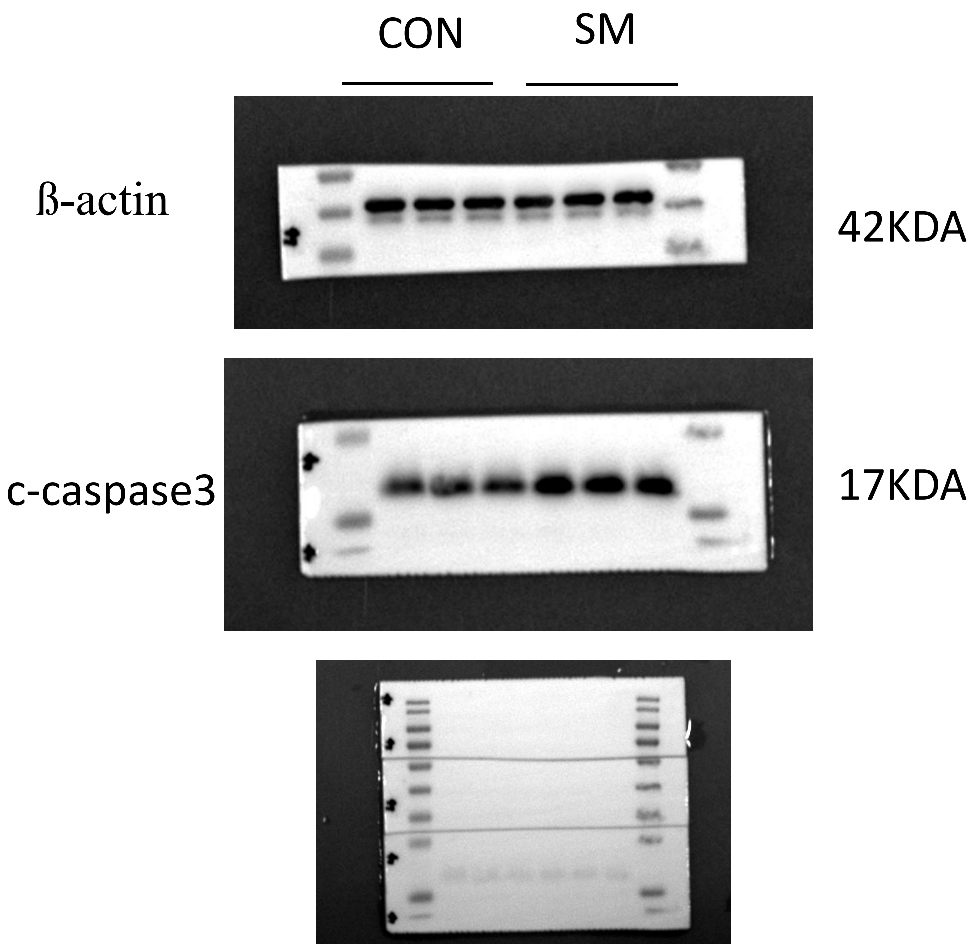
CON
SM
ß-actin
42KDA
c-caspase3
17KDA

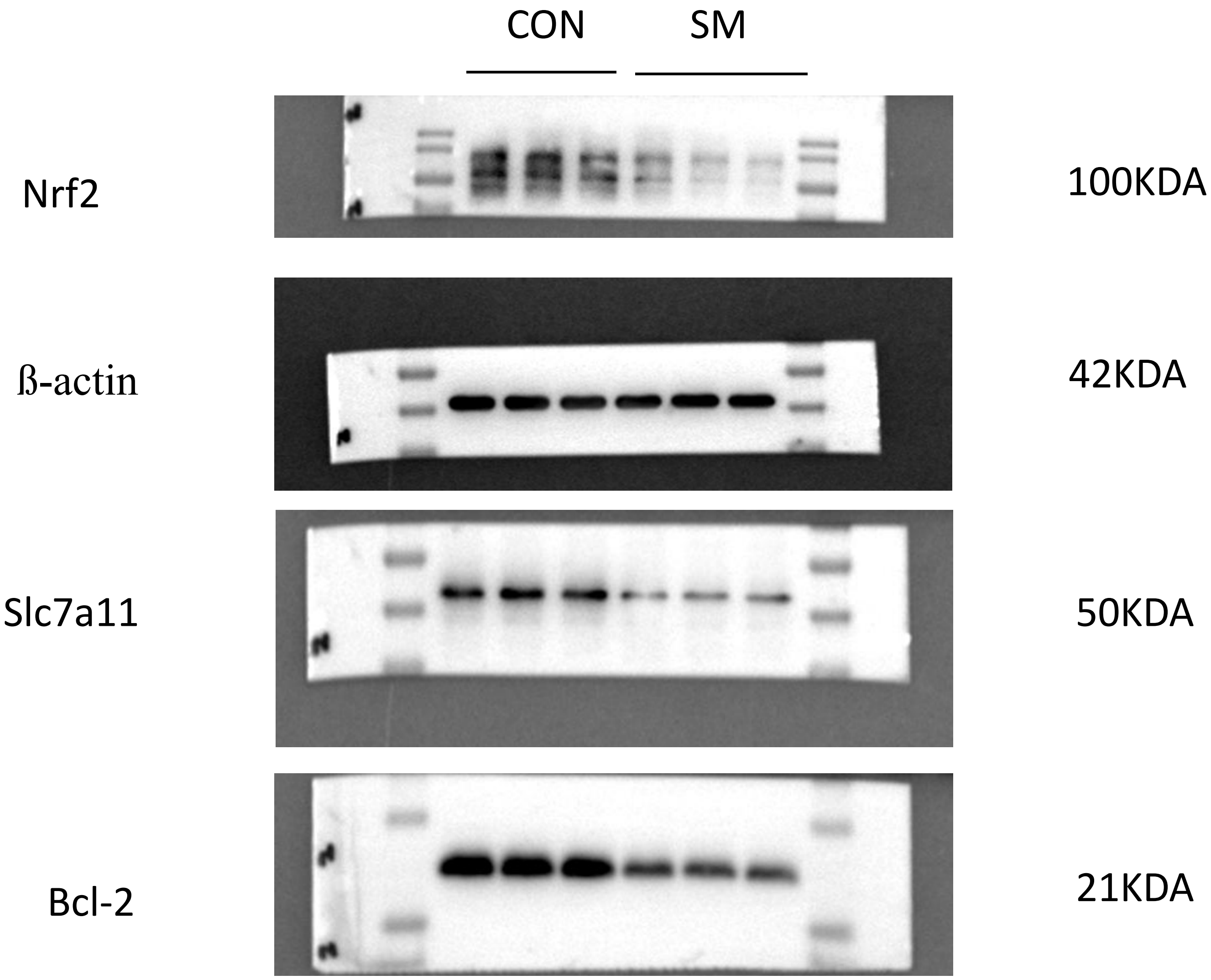
CON
SM
Nrf2
100KDA
ß-actin
42KDA
Slc7a11
50KDA
Bcl-2
21KDA

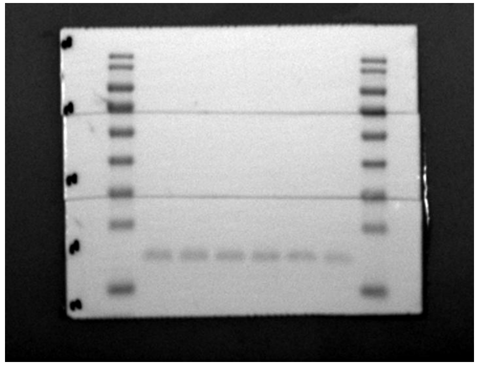

Supplement: Supplementary file 1 — Supplementary Information 1. [file 41598_2026_49458_MOESM1_ESM.pdf]
