# Supplementary material for: Solamargine induces apoptosis and ferroptosis through the ROS/p38 MAPK signalling pathway in intrahepatic cholangiocarcinoma
Source: Sci Rep. 2026 Apr 24;16:19045. doi: 10.1038/s41598-026-49458-3 (PMC13280501; doi:10.1038/s41598-026-49458-3)
Supplement: Supplementary file 2 — Supplementary Information 2. [file 41598_2026_49458_MOESM2_ESM.pdf]

HCCC-9810 细胞 STR 鉴定报告

一、材料处理和检测方法

取适量细胞（> 1×10⁶），使用 Tsingke 的动物基因组抽提试剂盒（货号 TSP201-200）提取细胞的基因组 DNA。使用新海生物的 NuHi SU9 dNTP Mix（货号 NH9347）对 19 个 STR 位点以及性别位点 Amelogenin 进行扩增。使用 Cellosaurus STR 相似度搜索工具 CLASTR 对检测数据进行分析，同时与 ATCC，DSMZ，JCRB 和 RIKEN 等多个细胞库以及文献资料进行比对。

二、检验结果

样品编号	多等位基因	匹配细胞系	细胞库	匹配度	匹配说明
HCCC-9810	无	HCCC-9810	CCRID	1.00	完全匹配

- 多等位基因指三等位及以上基因现象。

三、鉴定结论：

1、本次检测各细胞分型结果良好。

2、该细胞株 DNA 分型在细胞库中匹配到与其细胞分型 **100%**相匹配的细胞株，细胞株名为：**HCCC-9810**。

备注：

1、根据国际细胞鉴定委员会（ICLAC）制定的细胞 STR 鉴定标准，当细胞系匹配度为 1.00 时是完全匹配；匹配度在 0.80 到 0.99 之间时认定检测细胞与细胞库里匹配到的细胞具有相关性；但当匹配度小于 0.80 时表明检测细胞与细胞库里匹配到的细胞 STR 位点差异较大，两者之间没有关联，不能对检测细胞进行鉴定。

2、STR 比对结果默认 ExPASy，服务器公开访问地址（https://web.expasy.org/cellosaurus-str-search）。

附表：HCCC-9810 细胞的 STR 位点和 Amelogenin 位点的基因分型结果

细胞 HCCC-9810 的 STR 位点和 Amelogenin 位点的基因分型结果						
	送检细胞 STR 信息			细胞库细胞 STR 信息		
	送检细胞名：HCCC-9810			细胞库细胞名：HCCC-9810		
Loci	Allele1	Allele2	Allele3	Allele1	Allele2	Allele3
D5S818	11			11		
D13S317	11			11		
D7S820	10	12		10	12	
D16S539	9	12		9	12	
VWA	16	17		16	17	
TH01	9			9		
AMEL	X			X		
TPOX	8	11		8	11	
CSF1PO	11	12		11	12	
D12S391	19					
FGA	23	24		23	24	
D2S1338	17	19		17	19	
D21S11	30	32		30	32	
D18S51	15			15		
D8S1179	10	13		10	13	
D3S1358	17			17		
D6S1043	11	19				
PENTAE	19					
D19S433	13	14		13	14	
PENTAD	9	13				

鉴定日期：2022-09-21

梓杉生物

附图：**HCCC-9810** 细胞的 **STR** 位点和 **Amelogenin** 位点的基因分型结果

Applied Biosystems
GeneMapper Software 5

0921-1

Sample File	Sample Name	Panel	Marker	OS
HCCC-9810_Z1_A01.fsa	01	1Z	01-D5S818	

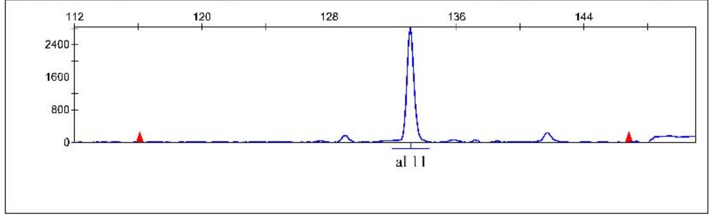

Sample File	Sample Name	Panel	Marker	OS
HCCC-9810_Z2_A05.fsa	33	2Z	02-D13S317	

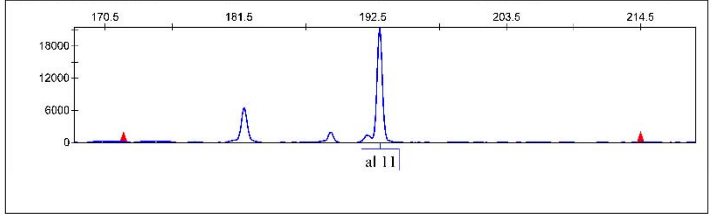

Sample File	Sample Name	Panel	Marker	OS
HCCC-9810_Z1_A01.fsa	01	1Z	03-D7S820	

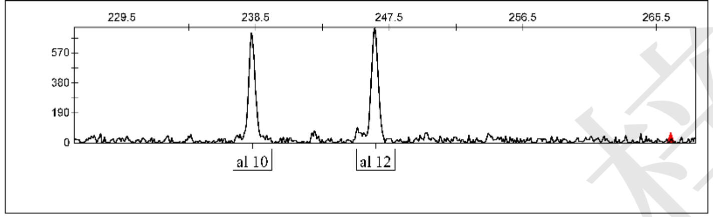

Wed Sep 21,2022 09:06AM, CST   Printed by: gm   Page 1 of 7

Applied Biosystems
GeneMapper Software 5

0921-1

Sample File	Sample Name	Panel	Marker	OS
HCCC-9810_Z1_A01.fsa	01	1Z	04-D16S539	

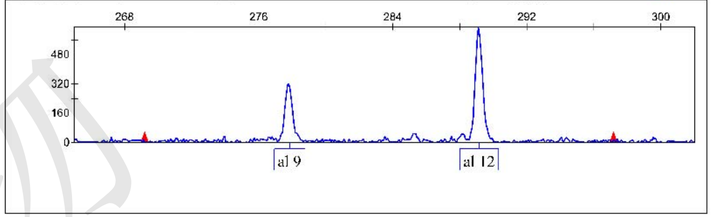

Sample File	Sample Name	Panel	Marker	OS
HCCC-9810_Z1_A01.fsa	01	1Z	05-VWA	

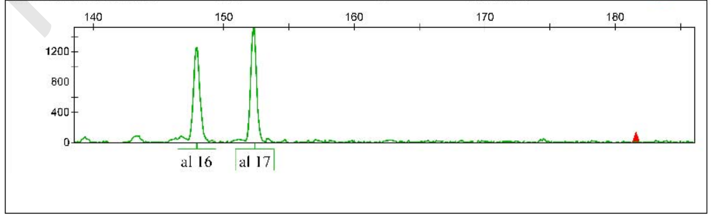

Sample File	Sample Name	Panel	Marker	OS
HCCC-9810_Z2_A05.fsa	33	2Z	06-TH01	

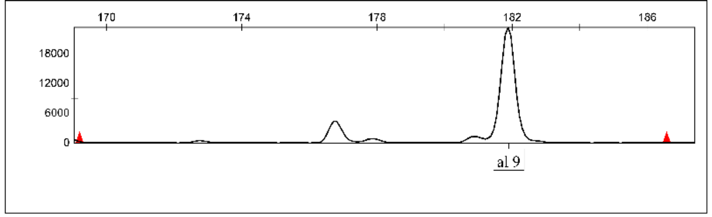

Wed Sep 21,2022 09:06AM, CST   Printed by: gm   Page 2 of 7

梓杉生物

Applied Biosystems
GeneMapper Software 5

0921-1

Sample File	Sample Name	Panel	Marker	OS
07_HCCC-9810_A01.fsa	01	07-AMEL	07-AMEL	

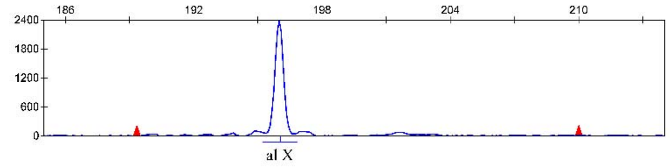

HCCC-9810_Z1_A01.fsa	01	1Z	08-TOPX	-4.0

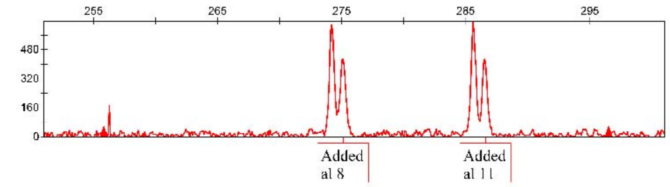

09_HCCC-9810_A05.fsa	33	09-CSF1PO	09-CSF1PO	

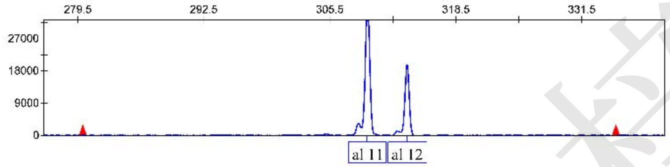

Wed Sep 21,2022 09:06AM, CST Printed by: gm Page 3 of 7

梓杉生物

Applied Biosystems
GeneMapper Software 5

0921-1

Sample File	Sample Name	Panel	Marker	OS
10_HCCC-9810_A09.fsa	65	10-D12S391	10-D12S391	

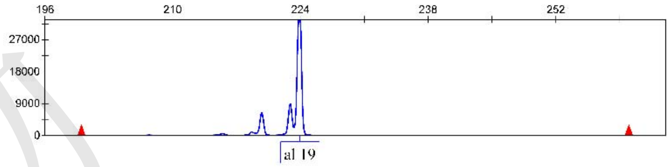

HCCC-9810_Z2_A05.fsa	33	2Z	11-FGA	

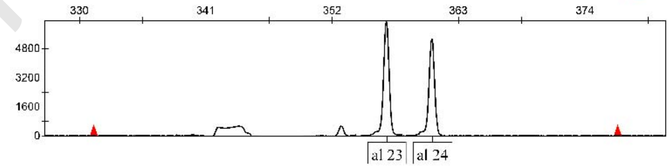

HCCC-9810_Z2_A05.fsa	33	2Z	12-D2S1338	

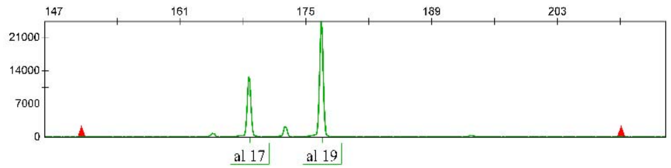

Wed Sep 21,2022 09:06AM, CST Printed by: gm Page 4 of 7

梓杉生物

武汉梓杉生物技术有限公司
地址：湖北省武汉市东湖高新技术开发区高新二路388号生物医药加速器22栋5楼
电话：027-85714038 网址：www.servicebio.cn 全国免费热线：4006027178

武汉梓杉生物技术有限公司
地址：湖北省武汉市东湖高新技术开发区高新二路388号生物医药加速器22栋5楼
电话：027-85714038 网址：www.servicebio.cn 全国免费热线：4006027178

Applied Biosystems 0921-1

GeneMapper Software 5

Sample File	Sample Name	Panel	Marker	OS
HCCC-9810 Z2 A05.fsa	33	2Z	13-D21S11	

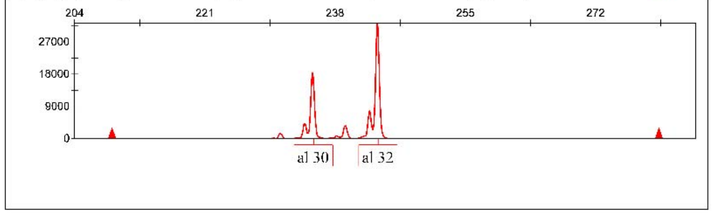

Sample File	Sample Name	Panel	Marker	OS
HCCC-9810 Z1 A01.fsa	01	1Z	14-D18S51	

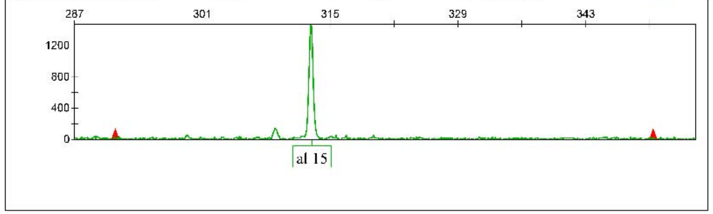

Sample File	Sample Name	Panel	Marker	OS
HCCC-9810 Z1 A01.fsa	01	1Z	15-D8S1179	

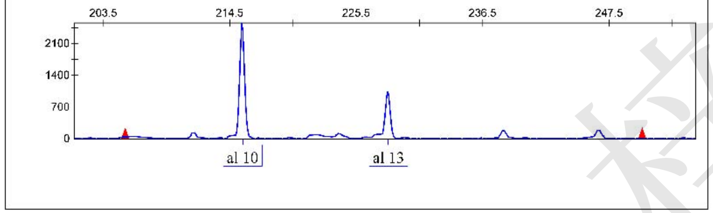

Wed Sep 21,2022 09:06AM, CST Printed by: gm Page 5 of 7

梓杉生物

Applied Biosystems 0921-1

GeneMapper Software 5

Sample File	Sample Name	Panel	Marker	OS
HCCC-9810 Z1 A01.fsa	01	1Z	16-D3S1358	-4.0

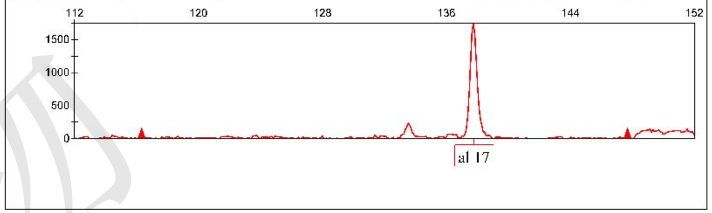

Sample File	Sample Name	Panel	Marker	OS
17 HCCC-9810 A09.fsa	65	17-D6S1043	17-D6S1043	

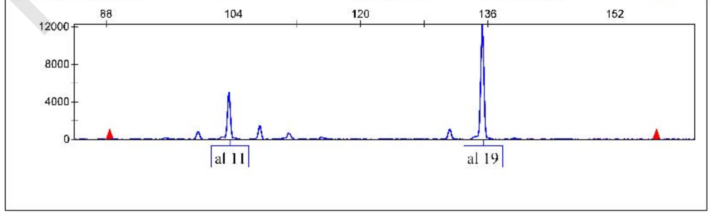

Sample File	Sample Name	Panel	Marker	OS
HCCC-9810 Z2 A05.fsa	33	2Z	18-PENTAE	

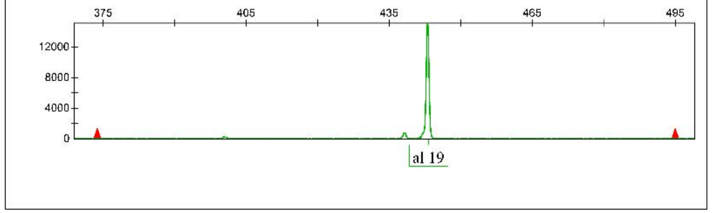

Wed Sep 21,2022 09:06AM, CST Printed by: gm Page 6 of 7

武汉梓杉生物技术有限公司
地址：湖北省武汉市东湖高新技术开发区高新二路388号生物医药加速器22栋5楼
电话：027-85714038 网址：www.servicebio.cn 全国免费热线：4006027178

梓杉生物

Applied Biosystems

GeneMapper Software 5

0921-1

Sample File	Sample Name	Panel	Marker	OS
HCCC-9810_Z1_A01.fsa	01	1Z	19-D19S433	

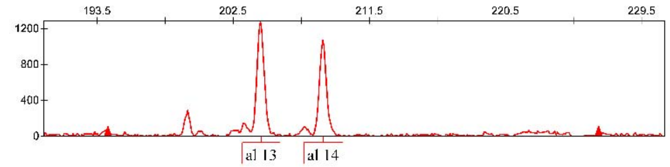

Sample File	Sample Name	Panel	Marker	OS
HCCC-9810_Z2_A05.fsa	33	2Z	20-PENTAD	

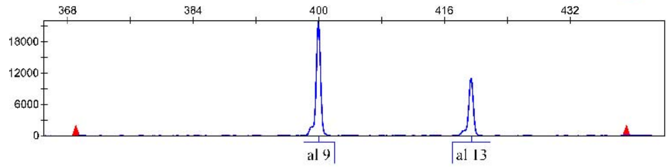

Wed Sep 21,2022 09:06AM, CST Printed by: gm Page 7 of 7

武汉梓杉生物技术有限公司
地址：湖北省武汉市东湖高新技术开发区高新二路388号生物医药加速器22栋5楼
电话：027-85714038 网址：www.servicebio.cn 全国免费热线：4006027178

梓杉生物

梓杉生物

武汉梓杉生物技术有限公司
地址：湖北省武汉市东湖高新技术开发区高新二路388号生物医药加速器22栋5楼
电话：027-85714038 网址：www.servicebio.cn 全国免费热线：4006027178

细胞 STR 检测报告

Cell Line STR Authentication Report

检测报告编号：20240808-06

细胞名称： HIBEC

送检日期：2024.8.2

样品数量：1

样品类型：细胞沉淀

样品处理：细胞沉淀（>1x10^6cells）用 PureLink™ Genomic DNA Mini Kit（Thermofisher K182001）提取基因组 DNA，使用 PowerPlex®18D 系统（美国 Promega DC1802）试剂盒进行基因扩增，通过 ABI 3730xl 型遗传分析仪对 PCR 产物进行检测。

检测结果:

<1>细胞 DNA 分型结果在 DSMZ 细胞库进行匹配，未找到匹配（EV 值大于0.8）的细胞。未发现多等位基因现象，排除人源细胞交叉污染。

<2>该细胞检测图谱清晰，分型结果良好。

<3>本次检测阴性及阳性对照结果正确。

附表 1： HIBEC细胞 STR 分型结果及匹配其细胞库信息

Locus	送检细胞名： HIBEC			细胞库细胞名：无		
D5S818	9	11				
D13S317	8	12				
D7S820	10	12				
D16S539	9	12				
VWA	17	17				
TH01	9.3	9.3				
AMEL	X	Y				
TPOX	8	8				
CSF1PO	10	12				
D12S391	18	20				
FGA	22	22				
D2S1338	17	17				
D21S11	30	30				
D18S51	14	15				
D8S1179	8	13				
D3S1358	15	15				
D6S1043	12	13				
PENTAE	15	17				
D19S433	12	14				
PENTAD	9	16				
D1S1656	18.3	18.3				

附图 1.细胞基因分型图谱

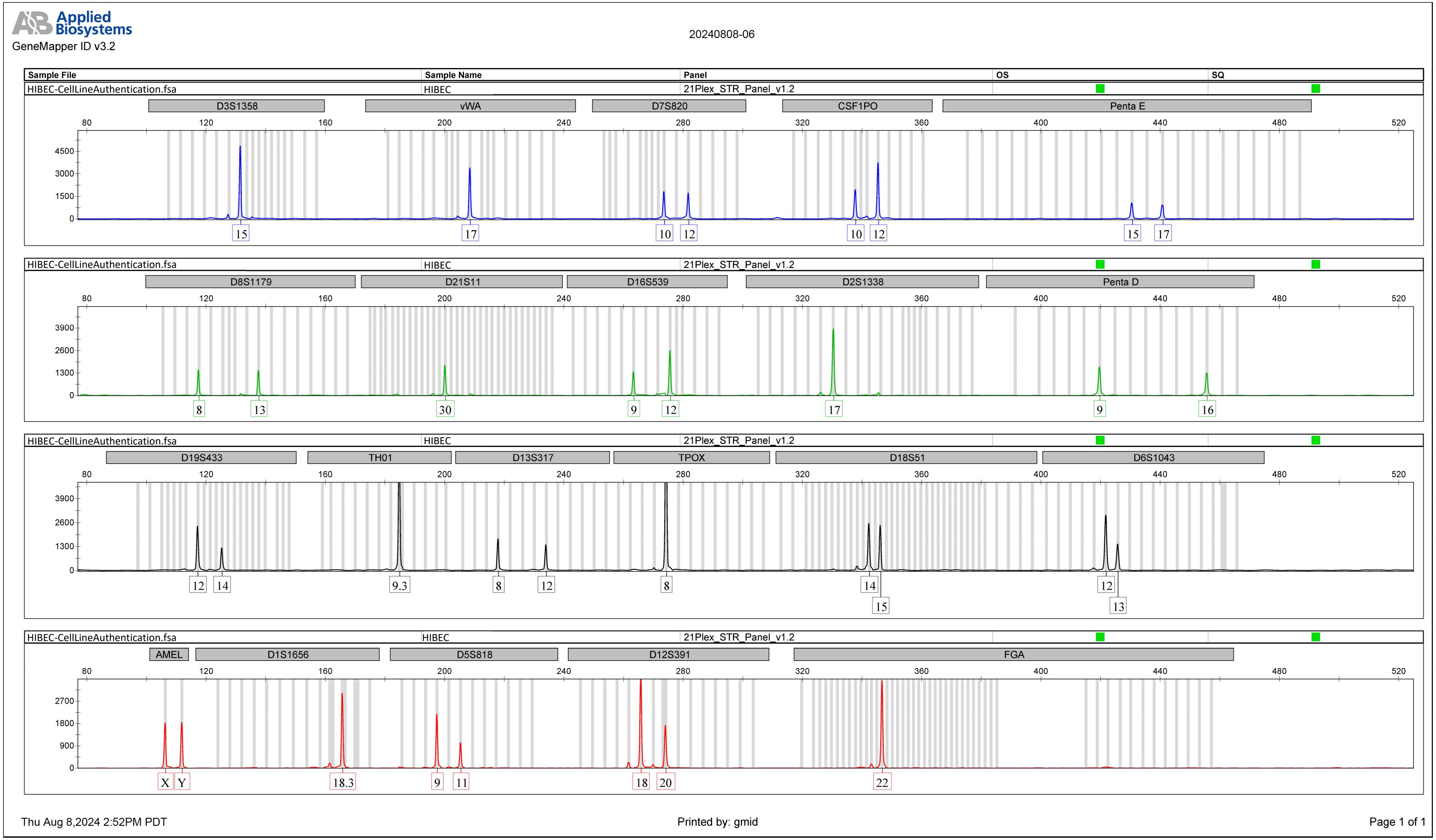

附图 2. STR 位点和 Amelogenin 位点 细胞库匹配结果

Refine search　　Start new search

ⓘ Your search uses the non-empty-based scoring mode.

Similarity	Cell line	Source	Shared	D5S818	D7S820	D13S317	D16S539	vWA	TH01	TPOX	CSF1PO	Amelogenin
		Your query		9, 11	10, 12	8, 12	9, 12	17, 17	9.3, 9.3	8, 8	10, 12	X, Y
77.8 %	M059J	CRL-2366	9	11, **12**	10, 12	**14**, **14**	**10**, 12	17, 17	9.3, 9.3	8, 8	10, 12	X, Y
72.2 %	NCI-H1284	CRL-5861	9	11, **12**	**9**, 10, **11**	8, 12	12, **13**	17, **19**	9.3, 9.3	8, **10**	10, 12	X, Y
72.2 %	M059K	CRL-2365	9	11, **12**	10, **10**	**14**, **14**	**10**, 12	17, 17	9.3, 9.3	8, 8	10, 12	X, Y
66.7 %	SU-DHL-7	DSMZ: ACC-903	9	11, **13**	10, **11**	12, **13**	9, 12	**16**, 17	**7**, 9.3	8, 8	10, 12	X, **X**
66.7 %	WA01	USNIH010	9	9, 11	**8**, 12	8, **11**	9, **13**	**15**, 17	9.3, 9.3	8, **11**	12, **13**	X, Y
66.7 %	ACJ Cells No. 36	STRJ0036	9	9, **10**	**9**, 12	**13**, **13**	9, **9**	17, 17	9.3, 9.3	8, 8	**11**, 12	X, Y
66.7 %	ACJ Cells No. 19	STRJ0009	9	9, **10**	**9**, 12	**13**, **13**	9, **9**	17, 17	9.3, 9.3	8, 8	**11**, 12	X, Y
66.7 %	MEC	RCB2129	9	11, **13**	10, **11**	8, **11**	9, 12	17, **18**	9.3, 9.3	8, **9**	10, **14**	X, Y
66.7 %	LoVo	RCB1639	9	11, **12**, **13**	10, **11**	8, **11**	9, 12	17, **18**	9.3, 9.3	8, **9**	**11**, 12, **13**, **14**	X, Y
66.7 %	SKW-3	RCB1168	9	**12**, **13**	**8**, 12	8, 12	**11**, 12	17, **18**	**6**, 9.3	8, 8	10, 12	X, Y
66.7 %	AT-PDH1TKB	RCB0546	9	11, **12**	**8**, 12	8, 12	9, 12	**16**, 17	**6**, **7**	8, 8	**11**, 12	X, Y

STR 匹配结果说明：

1. 根据国际细胞鉴定委员会(ICLAC)制定的细胞STR鉴定标准，细胞系的匹配度≥80% 时，认为它们具有相关性，即衍生于共同的祖先细胞；匹配度在55%至 80% 之间，需要进一步验证相关性；小于55%时，两者不具有相关性。
2. 默认采用DSMZ tools或ATCC tools与细胞库细胞比对，未收录于DSMZ或ATCC等大型细胞库的细胞将无法比对到目的细胞，有其他指定细胞库的请标注细胞库信息。
3. 为了保护细胞及细胞供者隐私，请勿公开所有STR位点信息。如需比对细胞库，提供DSMZ细胞库所需8个位点和性别位点信息即可。

细胞遗传质量鉴定检测

Cell Line Authentication Service

STR 基因型检测报告

送检单位：上海银海圣生物科技有限公司

检品名称：细胞系

检测单位：上海翼和应用生物技术有限公司

报告日期：2025-09-29

报 告 说 明

1. 本报告只对送检的来样负责。
2. 检验报告上的检验结果和检验单位名称，未经同意不得用于广告、评优及商业宣传。
3. 对本报告有异议，请于收到报告之日起十五日内以书面方式提出，逾期不予受理。
4. 对纸质检验报告涂改、增删，或未加盖检验单位印章的复印件均无效。

样 品 信 息

样品编号：

客户样本编号	公司编号
HuCC-T1	20250926-15

样品数量： 1

样品性状： 细胞系

检测项目： STR

送检单位： **上海银海圣生物科技有限公司**

检测方法： 用 Axygen 的基因组抽提试剂盒提取 DNA，采用 21- STR 扩增方案扩增，在 ABI 3730XL 型遗传分析仪上对 STR 位点和性别基因 Amelogenin 进行检测。

检测结果

(一) 检验基本情况

公司编号	多等位基因	匹配细胞系	细胞库	EV 值	匹配说明
20250926-15	无	HuCCT1	DSMZ	1.0	完全匹配

样本基因型检验结果

- 多等位基因指三等位及以上基因现象。
- 本次检测各细胞分型结果良好。

(二) 各样本描述

- 20250926-15：该株细胞 DNA 分型在细胞系检索中找到**完全匹配**的细胞系，DSMZ 数据库显示细胞名为 **HuCCT1**，细胞号对应 **JCRB0425**。本次检测在该细胞系中**没有发现多等位基因**。

  备注：待测细胞系与收录于 ATCC, DSMZ（DSMZ 收录了来自 ATCC、 DSMZ、JCRB 和 RIKEN 等 2490 株细胞的 STR 数据）, ExPASy 细胞库（ExPASy 收录了来自于 ATCC、DSMZ、JCRB、ECACC 和 Riken 等数据库约 8,000 株人源细胞 STR 数据）中的 STR 数据匹配，未收录于上述细胞库的细胞将无法匹配。根据 ATCC 标准委员会鉴定标准（ANSI/ATCC ASN-0002-2022）,匹配度 EV≥80%认为它们具有相关性，可能衍生于共同的祖先细胞；匹配度 55%-80%之间，需要结合其它方法进一步的鉴定认证其相关性。

（三）样本分型结果

细胞 20250926-15 的 STR 位点和 Amelogenin 位点的基因分型结果						
Loci	送检细胞 STR 信息			细胞库细胞 STR 信息		
	送检细胞名：HuCC-T1			细胞库细胞名：HuCCT1		
	Allele1	Allele2	Allele3	Allele1	Allele2	Allele3
D5S818	12	13		12	13	
D13S317	11	13		11	13	
D7S820	10	11		10	11	
D16S539	11	12		11	12	
VWA	18	18		18	18	
TH01	7	10		7	10	
AMEL	X	Y		X	Y	
TPOX	8	8		8	8	
CSF1PO	11	12		11	12	
D12S391	18	20				
FGA	20	23				
D2S1338	17	18				
D21S11	31	31				
D18S51	13	13				
D8S1179	10	10				
D3S1358	15	15				
D6S1043	13	13				
PENTAE	15	18				
D19S433	13	13				
PENTAD	10	10				
D1S1656	13	16				

其他说明

(一) 分型方案及位点分布

	方案 1	方案 2	方案 3	方案 4
1	D3S1358	D8S1179	D19S433	AMEL
2	VWA	D21S11	TH01	D1S1656
3	D7S820	D16S539	D13S317	D5S818
4	CSF1PO	D2S1338	TPOX	D12S391
5	PENTAE	PENTAD	D18S51	FGA
6			D6S1043	

实验方案及位点

(二) STR 数据库比对

本公司采用 DSMZ tools 进行细胞系比对，其中包含来自于 ATCC, DSMZ, JCRB 和 RIKEN 数据库的 2455 个细胞系 STR 数据。如果待检测细胞未收录于以上细胞库或这是自行建立的新细胞系将无法进行比对，用户需根据细胞分型结果自行与其他数据库进行比对。

(三) 文献引用参考

ATCC SDO. 2011. ASN-0002. Authentication of Human Cell Lines: Standardization of STR Profi ling. ANSI eStandards Store, by ATCC-Standards Development Organization (SDO). https://webstore.ansi.org/standards/atcc/ansiatccasn00022011. Accessed: September, 2021.

主要实验人员：陈静
复核人：何秀川
负责人：王敏

签发日期：2025-09-29

细胞遗传质量鉴定检测

Cell Line Authentication Service

STR 基因型检测报告

样 品 信 息

样品编号：

客户样本编号	公司编号
SB-1	20230310-01

样品数量： 1

样品性状： 细胞系

检测项目： STR

送检单位： 雅吉

检测方法： 用 Axygen 的基因组抽提试剂盒提取 DNA，采用 10- STR 扩增方案扩增，在 ABI 3730XL 型遗传分析仪上对 STR 位点和性别基因 Amelogenin 进行检测。

检 测 结 果

(一)　检验基本情况

编号	多等位基因	匹配细胞系	人源污染	与对比细胞匹配度 EV 值	匹配说明

20230310-01	有	鼠源细胞系	无	无

样本基因型检验结果

- 多等位基因指三等位及以上基因现象。
- 本次检测各细胞分型结果良好。

(二) 各样本描述

20230310-01：该株细胞鉴定结果为**小鼠细胞系**，DNA 分型在细胞系检索中未找到匹配的细胞系。本次检测在该细胞系中**发现多等位基因**。（因数据库未登录小鼠细胞系 **SB-1** 相关 STR 数据信息，无法显示匹配结果，若为发表论文可提交该数据给杂志即可）

（三）备注：待测细胞系与收录于 ATCC, DSMZ, JCRB 和 RIKEN 数据库的细胞系 STR 数据进行比对，未收录于以上细胞库的细胞系将无法匹配。

下列位点中 D4S2408 为人源位点，用于检测该细胞是否有人源污染。

（四）样本分型结果

细胞 20230310-01 的 STR 位点和 Amelogenin 位点的基因分型结果

Loci	送检细胞 STR 信息			细胞库细胞 STR 信息		
	送检细胞名：SB-1			细胞库细胞名：		
	Allele1	Allele2	Allele3	Allele1	Allele2	Allele3
4-2	233.91	241.94				
5-5	327.73	335.79				
6-4	290.72	294.71				
6-7	334.44	338.45	342.53			
9-2	221.44					
12-1	225.89	229.99				
15-3	188.99	192.96				
18-3	156.23					
X-1	396.93	413.29				

其他说明

(一) 分型方案及位点分布

	方案 1	方案 2
1	18-3(FAM)	12-1(FAM)
2	4-2 (FAM)	5-5(FAM)
3	6-7(FAM)	X-1(FAM)
4	9-2(NED)	15-3(NED)
5		6-4(NED)
6		D4S2408(NED)

实验方案及位点
