# Supplementary material for: Solamargine induces apoptosis and ferroptosis through the ROS/p38 MAPK signalling pathway in intrahepatic cholangiocarcinoma
Source: Sci Rep. 2026 Apr 24;16:19045. doi: 10.1038/s41598-026-49458-3 (PMC13280501; doi:10.1038/s41598-026-49458-3)
Supplement: Supplementary file 3 — Supplementary Information 3. [file 41598_2026_49458_MOESM3_ESM.pptx]

## Slide 1
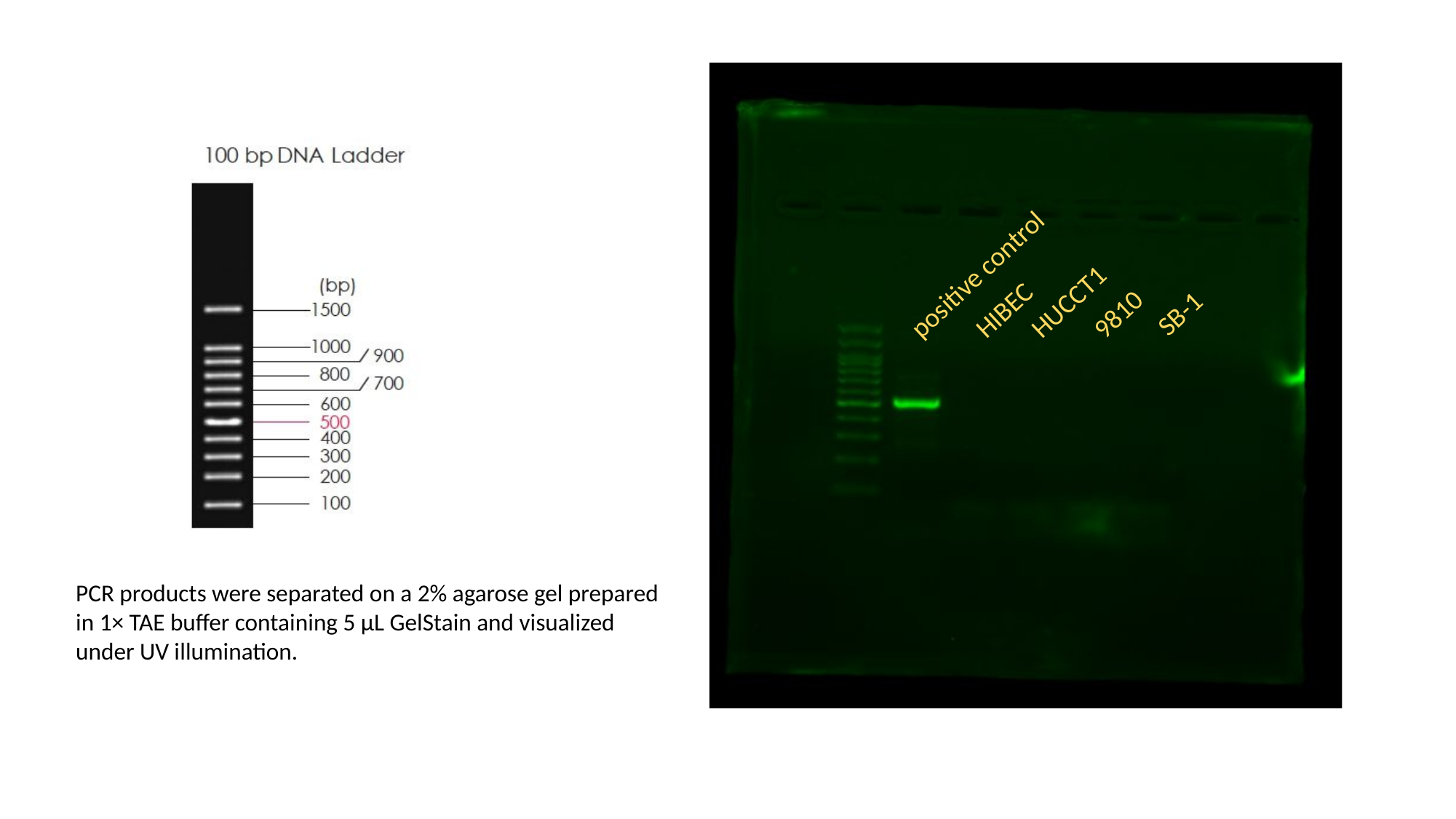

positive control
HUCCT1
9810
HIBEC
SB-1
PCR products were separated on a 2% agarose gel prepared in 1× TAE buffer containing 5 μL GelStain and visualized under UV illumination.
